# Associations of Estimated Glucose Disposal Rate With Stroke Risk and Poststroke Adverse Outcomes: A Prospective Cohort Study

**DOI:** 10.1111/cns.70420

**Published:** 2025-06-05

**Authors:** Xiaxuan Huang, Peina Dong, Yixian Xu, Yitong Ling, Shanyuan Tan, Zihong Bai, Si Shen, Jun Lyu, Hao Wang

**Affiliations:** ^1^ Department of Anesthesiology The First Affiliated Hospital of Jinan University Guangzhou China; ^2^ Department of Neurology The First Affiliated Hospital of Jinan University Guangzhou China; ^3^ Medical Imaging Center The First Affiliated Hospital of Jinan University Guangzhou Guangdong Province China; ^4^ Department of Clinical Research The First Affiliated Hospital of Jinan University Guangzhou China

**Keywords:** delirium, depression, disability, epilepsy, estimated glucose disposal rate, stroke

## Abstract

**Aims:**

This study investigated the relationship between estimated glucose disposal rate (eGDR), a validated marker of insulin resistance, and stroke subtypes and poststroke outcomes. Despite eGDR's established role in predicting cardiovascular outcomes, its impact on stroke risk and prognosis has not been fully explored.

**Methods:**

This study included 462,550 participants from the UK Biobank with eGDR assessments, and participants were stratified into three categories based on tertiles of eGDR. The primary outcomes were stroke and its subtypes (ischemic and hemorrhagic stroke). Cox proportional hazard models and restricted cubic spline regression were used to analyze associations between eGDR and outcomes. Secondary analyses investigated poststroke adverse events (depression, disability, epilepsy, and delirium). Mediation analyses were conducted to explore the underlying mechanisms driven by inflammatory markers, eGDR, and stroke.

**Results:**

During a median follow‐up of 13.9 years, 12,325 stroke cases were recorded. Compared to the lowest eGDR tertile (< 6.525 mg/kg/min), individuals in the highest tertile (> 8.494 mg/kg/min) demonstrated a significantly reduced risk of stroke (HR = 0.53, 95% CI: 0.50–0.56), particularly ischemic stroke (HR = 0.53, 95% CI: 0.50–0.57). Higher eGDR levels were also associated with a decreased risk of poststroke adverse outcomes (HR = 0.83, 95% CI: 0.73–0.94), with similar risk estimates observed for depression, disability, epilepsy, and delirium. Furthermore, inflammatory markers partially mediated the relationship between eGDR and stroke risk.

**Conclusions:**

Elevated eGDR levels were associated with decreased risks of stroke and poststroke adverse outcomes. These findings suggest improving insulin sensitivity, as reflected by higher eGDR, maybe a potential therapeutic target for stroke prevention or stroke rehabilitation.

## Graphical abstract

Leveraging UK Biobank data, this study investigated the associations between estimated glucose disposal rate (eGDR), an insulin resistance marker, and stroke outcomes. Analyses revealed longitudinal and nonlinear relationships between eGDR and stroke subtypes, poststroke complications, and identified inflammatory markers as mediators of eGDR's effect on stroke risk.

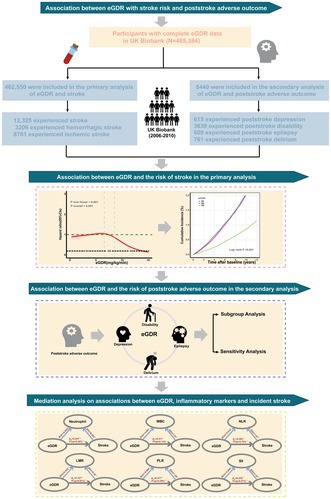

## 1 Introduction

Stroke, primarily characterized by acute focal neurological deficits, remains the second leading cause of death globally and the third leading cause of disability [1, 2]. Statistics indicate that by 2019, the global burden encompassed over 100 million survivors and 12 million new cases annually [3]. Among individuals who survive 6 months poststroke, nearly half require assistance with at least one activity of daily living. Beyond physical dependence and disability, the cumulative brain damage resulting from stroke leads to cognitive decline [4, 5]. Therefore, early identification of modifiable risk factors to prevent stroke and improve adverse poststroke outcomes represents an urgent public health priority.

Extensive epidemiological evidence has established hypertension, diabetes, hyperlipidemia, and obesity as principal modifiable risk factors for stroke [6, 7]. Current investigations increasingly focus on insulin resistance (IR) as a promising therapeutic target, particularly for stroke prevention in prediabetic populations. IR physiologically manifests through compensatory hyperinsulinemia that maintains normoglycemia despite diminished glucose uptake efficiency [8, 9, 10]. Although the hyperinsulinemic‐euglycemic clamp remains the gold standard for IR assessment, its clinical implementation is substantially constrained by procedural complexity, time requirements, cost considerations, and invasiveness [11]. Among various insulin resistance indices, the estimated glucose disposal rate (eGDR), derived from routinely collected clinical parameters, has gained significant clinical acceptance due to its demonstrated accuracy [12, 13]. This noninvasive, cost‐effective measurement integrates both clinical and laboratory indicators and has been validated against the hyperinsulinemic‐euglycemic clamp with high sensitivity, specificity, and precision in IR identification [14]. Consequently, eGDR has emerged as a clinically reliable surrogate marker for IR assessment in routine practice [15].

Recent evidence from a retrospective cohort analysis of the China National Stroke Registry III demonstrated that elevated eGDR values correlate with favorable functional outcomes in patients with first‐ever acute ischemic stroke (AIS) [16]. Despite this finding, significant knowledge gaps persist regarding the relationship between eGDR and various stroke subtypes. Additionally, while poststroke complications—including depression [17], disability [18], epilepsy [19], and delirium [20]—represent substantial clinical challenges, the predictive utility of eGDR for these adverse outcomes remains largely unexplored. Current evidence is primarily limited to AIS, highlighting the need for comprehensive research across different stroke subtypes and poststroke adverse outcomes. Moreover, previous studies have established clear associations between insulin resistance and endothelial dysfunction, hypercoagulability, and dyslipidemia—pathological processes exacerbated by low‐grade inflammation that accelerates atherogenesis and thrombosis [21]. Nevertheless, the relationship between eGDR‐associated inflammatory biomarkers and stroke risk remains inadequately characterized. Analysis of clinically accessible inflammatory markers could elucidate these mechanistic pathways and address this knowledge gap.

To expand upon this preliminary evidence, our study employed a large‐scale prospective investigation to overcome limitations in sample size and statistical power. Based on the UK Biobank's long‐term follow‐up and comprehensive covariate information, we conducted the first assessment of the relationship between eGDR, incident stroke risk, and poststroke outcomes (depression, disability, epilepsy, and delirium). In addition, this study leveraged blood marker data from the UK Biobank to explore potential mechanisms by which inflammatory markers may mediate the association between eGDR and stroke.

## 2 Methods

### 2.1 Study Population

The UK Biobank is a large‐scale prospective cohort study comprising 500,000 participants aged 37–73 years, recruited between 2006 and 2010 from 22 assessment centers across England, Scotland, and Wales [22, 23]. The research data were systematically collected through digital interfaces, personal interviews, and clinical evaluations, including health records and biological samples. This study was approved by the National Health Service North West Multi‐centre Research Ethics Committee. This manuscript adheres to the Strengthening the Reporting of Observational Studies in Epidemiology (STROBE) guidelines [24, 25].

**FIGURE 1:**
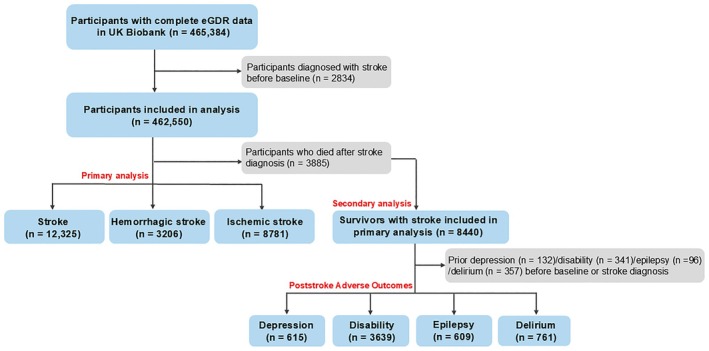
Flowchart of participants in the study.

Our study examined participants with complete eGDR data from the UK Biobank (*n* = 465,384). After excluding those diagnosed with stroke before baseline (*n* = 2834), we included 462,550 participants in the primary analysis. For the secondary analysis focusing on poststroke adverse outcomes, we identified participants who survived after stroke diagnosis (*n* = 8440), and we further excluded participants with pre‐existing conditions before baseline or stroke diagnosis: delirium (*n* = 357), epilepsy (*n* = 96), depression (*n* = 132), and disability (*n* = 341), as shown in Figure 1. These exclusions enabled independent assessment of each poststroke adverse outcome.

### 2.2 Exposure Measurements

Based on previous studies, the eGDR was calculated using the following formula [26]: eGDR (mg/kg/min) = 21.158 − (0.09 × WC) − (3.407 × HT) − (0.551 × HbA1c) [WC = waist circumference (cm), HTN = hypertension (yes = 1/no = 0), and HbA1c = glycosylated hemoglobin (%)]. The HbA1c values were determined by high‐performance liquid chromatography (HPLC) and converted to percentages [(0.09148 × HbA1c mmol/mol) + 2.152] [27]. Hypertension was defined as a diagnosis coded by the International Classification of Diseases, 10th Revision (ICD‐10), self‐reported history of hypertension, the use of antihypertensive medications, or an average systolic blood pressure (SBP) ≥ 140 mmHg or diastolic blood pressure (DBP) ≥ 90 mmHg measured at baseline [28]. Participants were categorized into eGDR tertiles, with the lowest tertile serving as the reference group.

### 2.3 Outcome Ascertainment

The primary outcome was incident stroke, identified through the International Classification of Diseases Tenth Revision (ICD‐10) codes, hospital admissions, self‐reported data, and primary care records (ICD‐10 codes H341, I63, I693, G46, I60, I61, I62, I690, I691, I692, I64, and I694), with subclassification into hemorrhagic stroke and ischemic stroke [29]. Secondary outcomes comprised poststroke adverse events: disability (including behavioral/emotional disorders, sensory impairments, physical limitations, and developmental disabilities; ICD‐10 codes Z736, Z74, F40‐F45, F50, F52, F54–F55, F59, F80, H53‐54, H90‐H91, R40‐R49, R53‐R54), depression/affective disorders (ICD‐10 codes F32‐34, F38‐39), epilepsy (ICD‐10 codes G40), and delirium (ICD‐10 codes F05). Follow‐up time was calculated from baseline to the first occurrence of stroke, poststroke outcomes, death, loss to follow‐up, or last documented clinical contact (Table S1).

### 2.4 Covariates and Mediators

Covariate selection was guided by directed acyclic graph (DAG) analysis based on previous literature [30]. This approach revealed complex variable relationships (Figure S1), allowing the classification of variables as confounders or mediators based on their hypothesized relationships with eGDR and stroke risk. Confounding variables were assessed at baseline from self‐reported data and medical records. Demographic factors included age, sex, and ethnicity (White/Ethnic minorities). Socioeconomic status was measured using the Townsend deprivation index (TDI) was self‐reported and categorized as low, intermediate, and high; educational attainment was self‐reported and categorized as low or high based on qualifications with or without a university or college degree level. Lifestyle factors encompassed self‐reported smoking status and alcohol intake status, which were categorized as never, previous, and current. Clinical measurements included systolic blood pressure (SBP) and diastolic blood pressure (DBP). Body mass index (BMI) was calculated by dividing weight (kg) by height (m) squared. Metabolic syndrome was defined as ≥ 3 of: central obesity, diabetes, hypertension, low HDL, and elevated triglycerides. A healthy diet required meeting ≥ 4 criteria: fruits (≥ 3 servings/day), vegetables (≥ 3/day), fish (≥ 2/week), processed meats (≤ 1/week), red meats (≤ 1.5/week), whole grains (≥ 3/day), and refined grains (≤ 1.5/day). Sleep duration was categorized as ≤ 6, 7–8, or ≥ 9 h/day. Potential co‐morbidities that may be associated with stroke and adverse stroke outcomes that were further focused on and considered in this study include the history of diabetes, hypercholesterolemia, hypertension, heart failure, and coronary heart disease based on ICD‐10 codes. Frailty was defined as meeting ≥ 3 of 5 criteria: weight loss, fatigue, low physical activity, slow walking speed, and low grip strength. Self‐reported medication use (aspirin, antihypertensive agents, lipid‐lowering drugs) was documented through verbal interviews (Supporting Methods).

Inflammatory markers were measured in baseline blood samples. The Beckman Coulter LH750 analyzer quantifies the total count of white blood cells and platelets, as well as the total count of immune cells including neutrophils, lymphocytes, monocytes, and their percentage in leukocytes, while C‐reactive protein is measured using the AU5800 immunoturbidimetric analyzer. Systemic inflammatory status was assessed through calculated ratios: neutrophil‐to‐lymphocyte (NLR), lymphocyte‐to‐monocyte (LMR), platelet‐to‐lymphocyte (PLR), and systemic immune‐inflammation index [SII = (neutrophils × platelets)/lymphocytes]. Based on the DAG analysis, these inflammatory markers were identified as potential mediators in the causal pathway between eGDR and stroke. All inflammatory markers underwent standardization, with log‐transformation for skewed distributions, followed by z‐score conversion (Table S2).

### 2.5 Statistical Analysis

The Shapiro–Wilk test was used to assess the normality of continuous variables. As all continuous variables showed a non‐normal distribution, they were expressed as median with interquartile range (IQR) and analyzed using the Kruskal–Wallis test for between‐group comparisons. Categorical variables were presented as counts(percentages) and compared using the chi‐square test. The participants were stratified into three groups based on the tertiles of eGDR: Q1 (eGDR < 6.525 mg/kg/min), Q2 (6.525 mg/kg/min ≤ eGDR ≤ 8.494 mg/kg/min), and Q3 (eGDR > 8.494 mg/kg/min). Missing covariates were handled using multiple imputation by chained equations (Table S3).

In the preliminary analysis, Kaplan–Meier survival curves were constructed to illustrate cumulative stroke incidence across eGDR tertiles, with differences in survival distributions evaluated using log‐rank tests. eGDR was analyzed as a continuous variable, and restricted cubic spline (RCS) regression was employed to model potential nonlinear relationships, with knots placed at the 5th, 35th, 65th, and 95th percentiles. The likelihood ratio test was utilized to evaluate the presence of nonlinearity. To assess the association between eGDR and the risks of all strokes, ischemic strokes, and hemorrhagic strokes, three progressively adjusted Cox proportional hazards models were constructed: Model 1 adjusted for age and sex at baseline; Model 2 additionally adjusted for ethnicity, TDI, and educational level; and Model 3, the primary model, further adjusted for smoking status, alcohol intake status, SBP, DBP, sleep duration, BMI, MetS, and healthy diet. Hazard ratios and 95% CIs were estimated to quantify the associations. Schoenfeld residuals confirmed the proportional hazards assumption, and variance inflation factors below 2 indicated acceptable multicollinearity (Table S4). Model selection utilized Akaike and Bayesian information criteria (Table S5). Schoenfeld residuals confirmed the proportional hazards assumption, and variance inflation factors below 2 indicated acceptable multicollinearity. The secondary analysis aimed to investigate the association between eGDR and poststroke adverse outcomes, including depression, disability, epilepsy, and delirium. To ensure that the analysis focused exclusively on incident adverse events following stroke, patients with pre‐existing conditions of these outcomes were excluded. Within the same analytical framework, Cox proportional hazard models were employed to separately evaluate the relationship between eGDR and each adverse outcome. To address the issue of low event rates for certain outcomes, a composite endpoint was constructed by integrating all adverse events, with the earliest occurrence of any event defining the endpoint.

To evaluate result robustness, we performed multiple sensitivity analyses. Further adjustments were made for potential covariates. Model 4 incorporated medication use (aspirin, antihypertensive, and lipid‐lowering agents), Model 5 accounted for comorbidities (hypertension, diabetes, hypercholesterolemia, frailty, heart failure, and coronary heart disease), and Model 6 combined all variables. Further sensitivity analyses excluded participants aged below 55 years to address limited follow‐up concerns, excluded those with less than 5 years of follow‐up to minimize reverse causation, and conducted separate analyses excluding individuals with ICD‐10‐diagnosed diabetes to validate associations in nondiabetic populations. The Fine and Gray competing risks model was employed to account for death as a competing risk factor in the analysis of longitudinal associations between eGDR and stroke incidence as well as poststroke outcomes, thus addressing potential biases. Additionally, a piecewise Cox model was applied to assess the risks of poststroke depression, disability, delirium, and epilepsy at three time points—3 months, 6 months, and 1 year—among stroke survivors. Subgroup analyses were stratified by age, sex, ethnicity, education, smoking status, alcohol intake, and obesity status (BMI < 30 /≥ 30). Effect modification was examined using multiplicative interaction terms for each covariate in the primary model.

Based on previous evidence, IR can impair glucose metabolism and induce a pro‐inflammatory state by reducing insulin sensitivity [31]. This study utilized multivariate linear regression to examine the association between inflammatory status and eGDR. Standardized beta coefficients were derived from a fully adjusted model and converted to Cohen's d [32], with Bonferroni correction applied for multiple comparisons. To assess the potential mediating role of inflammatory status in the eGDR–stroke risk relationship, a three‐variable mediation model was employed. The “mediation” package was used to fit a bootstrapped mediation model, evaluating whether inflammatory markers mediated the longitudinal association between eGDR and stroke risk. Linear regression models analyzed eGDR–inflammatory marker correlations, while survival regression models assessed associations of inflammatory markers and eGDR with stroke outcomes. Total effects (TEs) were partitioned into natural direct effects (NDEs) and natural indirect effects (NIEs), with mediation effects estimated via 1000 bootstrap iterations. The mediation proportion was calculated as NDE × (NIE − 1)/(TE − 1) [33]. All models incorporated the primary analysis covariates; the false discovery rate (FDR) was obtained using the Benjamini–Hochberg (BH) method to correct for multiple comparisons (Supporting Methods).

All P values were reported as two‐sided tests with significance defined as *p* < 0.05. Statistical analyses were performed in the R software (version 4.4.1).

## 3 Results

### 3.1 Baseline Characteristics

Table 1 presents baseline characteristics of participants stratified by eGDR tertiles. Among a total of 462,550 participants, the mean age was 58 years, with a predominance of females (54.5%). Compared to the lowest tertile (eGDR < 6.525 mg/kg/min), participants in the highest tertile (eGDR > 8.494 mg/kg/min) were younger, predominantly female, more likely to be never‐smokers, and had lower BMI and fewer comorbidities. Lower eGDR levels were associated with elevated inflammatory markers, including WBC count, CRP, SII, and NLR values.

**TABLE 1:** Baseline characteristics of 462,550 participants by tertile of eGDR in UK biobank study.

Characteristics	Total	eGDR (mg/kg/min)		
		Q1 (< 6.525)	Q2 (6.525–8.494)	Q3 (> 8.494)
*N*	462,550	154,185	154,181	154,184
Age, years	58 (50,63)	59 (51,64)	59 (51,64)	55 (48,62)
Sex, *n* %				
Female	252,060 (54.5)	78,779 (51.1)	82,701 (53.6)	90,580 (58.7)
Male	210,490 (45.5)	75,406 (48.9)	71,480 (46.4)	63,604 (41.3)
TDI category, *n* %				
Low	114,788 (24.8)	37,559 (24.4)	37,641 (24.4)	39,588 (25.7)
Medium	232,332 (50.2)	77,059 (50)	77,264 (50.1)	78,009 (50.6)
High	115,430 (25)	39,567 (25.7)	39,276 (25.5)	36,587 (23.7)
Ethnicity, *n* %				
Ethnic minorities	43,350 (9.4)	14,295 (9.3)	14,242 (9.2)	14,813 (9.6)
White	419,200 (90.6)	139,890 (90.7)	139,939 (90.8)	139,371 (90.4)
Education, *n* %				
Low level	200,786 (43.4)	68,208 (44.2)	68,546 (44.5)	64,032 (41.5)
High level	261,764 (56.6)	85,977 (55.8)	85,635 (55.5)	90,152 (58.5)
Smoking status, *n* %				
Never	253,937 (54.9)	82,200 (53.3)	82,866 (53.7)	88,871 (57.6)
Previous	159,734 (34.5)	55,800 (36.2)	54,914 (35.6)	49,020 (31.8)
Current	48,879 (10.6)	16,185 (10.5)	16,401 (10.6)	16,293 (10.6)
Alcohol intake status, *n* %				
Never	21,392 (4.6)	7390 (4.8)	7468 (4.8)	6534 (4.2)
Previous	16,522 (3.6)	5789 (3.8)	5850 (3.8)	4883 (3.2)
Current	424,636 (91.8)	141,006 (91.5)	140,863 (91.4)	142,767 (92.6)
Healthy diet, *n* %	424,292 (91.7)	141,329 (91.7)	141,748 (91.9)	141,215 (91.6)
Waist (cm)	90 (80,99)	100.5 (96,107)	84 (78,89)	83 (74,92)
BMI, (kg/m²)	26.7 (24.1,29.9)	27.3 (24.6,30.7)	27 (24.4,30.2)	25.9 (23.4,28.7)
SBP, (mmHg)	138 (125,151)	148 (138,159)	143 (130,155)	126 (117,133)
DBP, (mmHg)	82 (75,89)	87 (80,94)	83 (76,90)	76 (70,81)
Glucose, (mmol/L)	4.9 (4.6,5.3)	5 (4.6,5.4)	5 (4.6,5.3)	4.9 (4.6,5.2)
HDL, (mmol/L)	1.4 (1.2,1.7)	1.4 (1.1,1.6)	1.4 (1.2,1.7)	1.4 (1.2,1.7)
WBC, (10^9^ cells/L)	6.7 (5.6,7.8)	6.8 (5.7,8)	6.7 (5.7,7.9)	6.5 (5.5,7.7)
Platelet counts, (10^9^ cells/L)	248 (213.3,287)	247.3 (212.3,287)	248 (213,287.5)	248.5 (214.6,287)
Lymphocyte counts, (10^9^ cells/L)	1.9 (1.5,2.3)	1.9 (1.5,2.3)	1.9 (1.5,2.3)	1.9 (1.5,2.2)
Monocyte counts, (10^9^ cells/L)	0.5 (0.4,0.6)	0.5 (0.4,0.6)	0.4 (0.3,0.5)	0.5 (0.4,0.6)
Neutrophil counts, (10^9^ cells/L)	4.1 (3.3,5)	4.1 (3.3,5)	3.9 (3.2,4.8)	4.1 (3.3,5)
Lymphocyte percentage	28.6 (23.9,33.5)	28.4 (23.7,33.3)	28.4 (23.7,33.4)	28.9 (24.3,33.9)
Monocyte percentage	6.8 (5.6,8.2)	6.9 (5.6,8.3)	6.9 (5.6,8.3)	6.8 (5.5,8.2)
Neutrophil percentage	61.1 (55.6,66.5)	61.3 (55.8,66.6)	61.2 (55.7,66.6)	60.9 (55.4,66.2)
CRP, (mg/L)	1.3 (0.7,2.8)	1.4 (0.7,3)	1.4 (0.7,2.9)	1.1 (0.6,2.4)
NLR	2.1 (1.7,2.8)	2.2 (1.7,2.8)	2.2 (1.7,2.8)	2.1 (1.6,2.7)
LMR	4.2 (3.3,5.4)	4.1 (3.2,5.3)	4.2 (3.2,5.3)	4.3 (3.3,5.5)
PLR	8.7 (7,11)	8.8 (7,11.1)	8.8 (7,11.1)	8.6 (6.9,10.9)
SII	529.1 (392.5,716.3)	533.3 (394.6,723.5)	532.9 (394.7,722.6)	520.8 (388.4,702.7)
Sleep duration, (hours/day)	7 (7,8)	7 (6,8)	7 (6,8)	7 (7,8)
MetS, *n* %	142,063 (30.7)	81,256 (52.7)	47,621 (30.9)	13,186 (8.6)
Coronary heart disease, *n* %	55,862 (12.1)	18,680 (12.1)	18,634 (12.1)	18,548 (12)
Hyperlipidemia, *n* %	42,056 (9.1)	16,534 (10.7)	18,035 (11.7)	7487 (4.9)
Hypertension, *n* %	110,637 (23.9)	50,958 (33)	52,973 (34.4)	6706 (4.3)
Diabetes, *n* %	50,575 (10.9)	27,009 (17.5)	16,488 (10.7)	7078 (4.6)
Physical frailty, *n* %	20,113 (4.3)	7990 (5.2)	7787 (5.1)	4336 (2.8)
Aspirin, *n* %	60,485 (13.1)	24,944 (16.2)	24,246 (15.7)	11,295 (7.3)
Anti‐hypertensive drugs, *n* %	32,155 (7)	14,288 (9.3)	14,075 (9.1)	3792 (2.5)
Lipid lowering medications, *n* %	31,411 (6.8)	12,562 (8.1)	13,423 (8.7)	5426 (3.5)
Stroke, *n* %	12,325 (2.7)	5056 (3.3)	5107 (3.3)	2162 (1.4)
Ischemic stroke, *n* %	8781 (1.9)	3633 (2.4)	3659 (2.4)	1489 (1)
Hemorrhagic stroke, *n* %	3206 (0.7)	1262 (0.8)	1314 (0.9)	630 (0.4)

Abbreviations: BMI, body mass index; CRP C‐reactive protein; DBP, diastolic blood pressure; eGDR, Estimated glucose disposal rate; HDL, high‐density lipoprotein cholesterol; LMR, Lymphocytes‐to‐monocytes ratio; MetS, metabolic syndrome; NLR neutrophil‐to‐lymphocyte ratio, PLR, Platelets‐to‐lymphocytes ratio; WBC, White blood cell; SBP, systolic blood pressure; SII, Systemic Immune Inflammation Index; TDI, Townsend deprivation index.

### 3.2 Association Between eGDR and Incident Stroke

**FIGURE 2:**
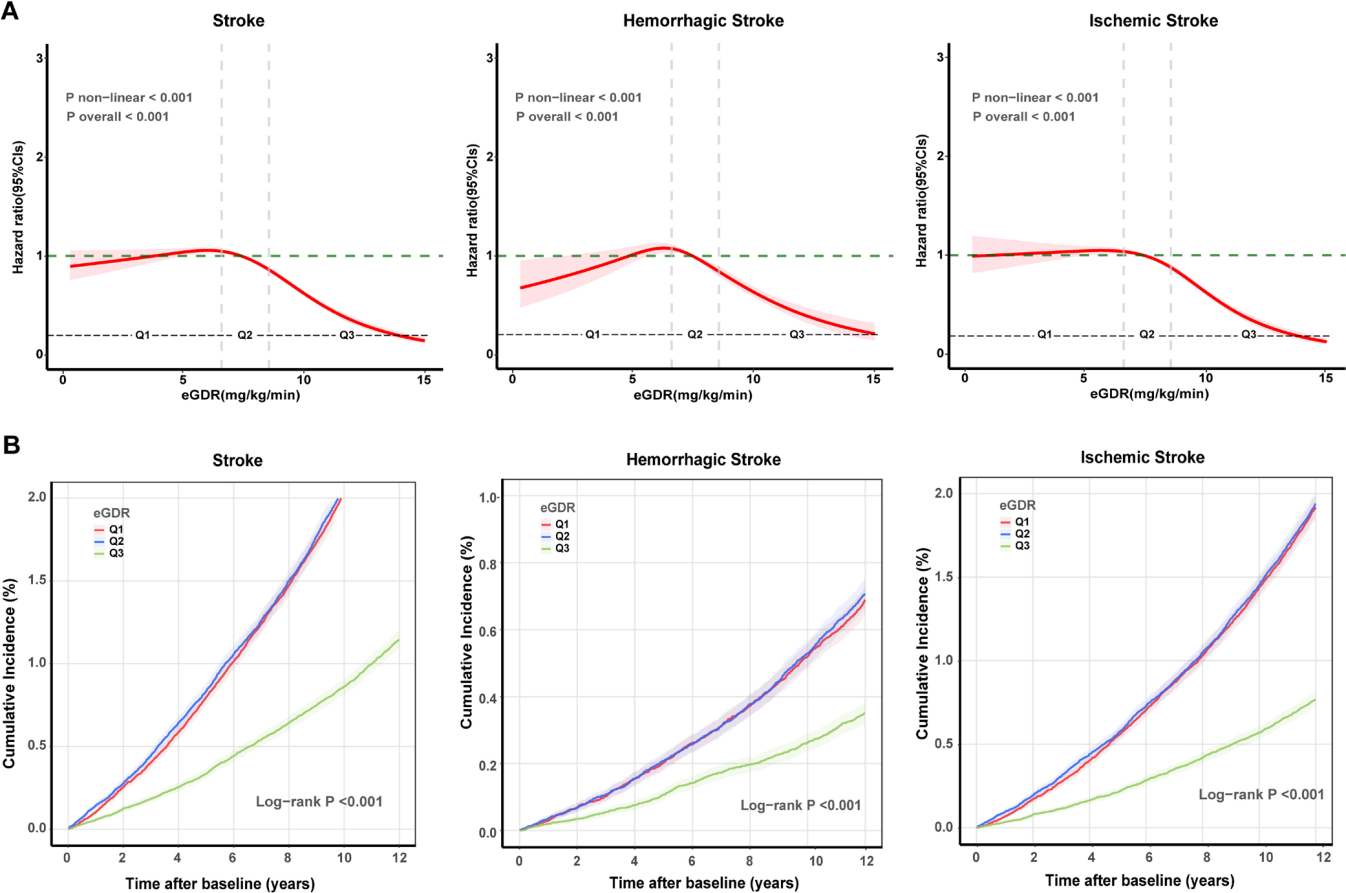
Association between eGDR level and the risk of stroke outcomes. (A) The dose–response associations between eGDR and stroke, ischemic stroke, and hemorrhagic stroke incidence. (B) Cumulative incidence of stroke, ischemic stroke, and hemorrhagic stroke according to baseline eGDR tertiles. *Note:* Nonlinear associations between eGDR and stroke outcomes were identified. The associations were further analyzed using eGDR tertiles. The hazard ratios for stroke risk are presented by eGDR tertiles, with the 1st tertile (lowest eGDR) as reference. Models adjusted for age, sex, ethnicity, education, Townsend deprivation index (TDI), smoking status, alcohol intake status, body mass index (BMI), systolic blood pressure (SBP), diastolic blood pressure (DBP), sleep duration, metabolic syndrome (MetS), and healthy diet.

During a median follow‐up of 13.9 years, we documented 12,325 stroke incidents (8781 ischemic, 3206 hemorrhagic stroke). In Table 2, after adjusting for potential confounders, including age, sex, ethnicity, TDI, education level, smoking status, alcohol intake status, BMI, SBP, DBP, sleep duration, MetS, and healthy diet, compared to the lowest eGDR tertile (< 6.525 mg/kg/min), the highest tertile demonstrated significantly reduced risks for developing stroke (HR = 0.53, 95% CI: 0.50–0.56), hemorrhagic stroke (HR = 0.55, 95% CI: 0.49–0.62), and ischemic stroke (HR = 0.53, 95% CI: 0.50–0.57). Consistent risk patterns were observed for eGDR measures when they were regressed as continuous variables (HR = 0.90, 95% CI: 0.89–0.91). Figure 2A shows that progressive risk reduction was observed across increasing eGDR levels, with the highest tertile showing a lower cumulative incidence for all stroke types. Furthermore, restricted cubic spline analysis revealed a nonlinear inverse relationship between eGDR levels and the risk of stroke, hemorrhagic stroke, and ischemic stroke incidents (Figure 2B, *p* for nonlinearity < 0.001).

**TABLE 2:** Association between eGDR and risk for incident stroke.

Outcome	Case/Person‐years	Model 1		Model 2		Model 3	
		HR (95% CI)	*p*	HR (95% CI)	*p*	HR (95% CI)	*p*
**Stroke**							
eGDR	12,325/6,363,081	0.89 (0.88–0.90)	< 0.001	0.90 (0.89–0.91)	< 0.001	0.90 (0.89–0.91)	< 0.001
Q1 (< 6.525)	5056/2,114,800	Reference					
Q2 (6.525–8.494)	5107/2,114,874	1.00 (0.96–1.04)	0.934	1.01 (0.98–1.05)	0.450	1.04 (0.99–1.08)	0.074
Q3 (> 8.494)	2162/2,133,407	0.50 (0.48–0.53)	< 0.001	0.53 (0.50–0.56)	< 0.001	0.53 (0.50–0.56)	< 0.001
**Hemorrhagic stroke**							
eGDR	3206/6,363,554	0.91 (0.89–0.92)	< 0.001	0.91 (0.89–0.93)	< 0.001	0.91 (0.89–0.93)	< 0.001
Q1 (< 6.525)	1262/2,114,958	Reference					
Q2 (6.525–8.494)	1314/2,115,102	1.03 (0.96–1.12)	0.402	1.04 (0.96–1.12)	0.299	1.03 (0.95–1.12)	0.432
Q3 (> 8.494)	630/2,133,493	0.58 (0.52–0.64)	< 0.001	0.59 (0.54–0.65)	< 0.001	0.55 (0.49–0.62)	< 0.001
**Ischemic stroke**							
eGDR	8781/6,363,634	0.88 (0.87–0.89)	< 0.001	0.89 (0.89–0.90)	< 0.001	0.90 (0.89–0.91)	< 0.001
Q1 (< 6.525)	3633/2,115,045	Reference					
Q2 (6.525–8.494)	3659/2,115,113	1.00 (0.95–1.04)	0.934	1.01 (0.97–1.06)	0.562	1.05 (0.99–1.10)	0.064
Q3 (> 8.494)	1489/2,133,477	0.49 (0.46–0.52)	< 0.001	0.52 (0.49–0.55)	< 0.001	0.53 (0.50–0.57)	< 0.001

*Note:* Model 1 adjusted for age, sex; Model 2 adjusted for age, sex, ethnicity, Townsend deprivation index (TDI), education; Model 3 adjusted for age, sex, ethnicity, TDI, education, smoking status, alcohol intake status, Body mass index (BMI), systolic blood pressure (SBP), diastolic blood pressure (DBP), sleep duration, metabolic syndrome (MetS) and healthy diet.

Abbreviations: CI, confidence interval; eGDR, estimated glucose disposal rate; HR, hazard ratio.

### 3.3 Association Between eGDR and Poststroke Adverse Outcomes

Among all stroke survivors, we observed a total of 2724 incident cases of poststroke adverse outcomes after excluding patients with prestroke or baseline diagnoses of depression, disability, epilepsy, and delirium. After fully adjusting for covariates and accounting for the time lag between stroke onset and baseline eGDR measurement, individuals in the highest eGDR tertile had a significantly reduced risk of all poststroke adverse outcomes compared to those in the lowest eGDR tertile (HR = 0.83, 95% CI: 0.73–0.94, *p* = 0.004). Specifically, as shown in Table 3, compared to the lowest eGDR tertile, individuals in the highest eGDR tertile had a 34% lower risk of poststroke depression (HR = 0.66, 95% CI: 0.47–0.94, *p* = 0.022), a 16% lower risk of poststroke disability (HR = 0.84, 95% CI: 0.74–0.96, *p* = 0.011), a 39% lower risk of poststroke epilepsy (HR = 0.61, 95% CI: 0.42–0.89, *p* = 0.010), and a 43% lower risk of poststroke delirium (HR = 0.57, 95% CI: 0.38–0.86, *p* = 0.007). Notably, the risk reductions were most pronounced for poststroke delirium and epilepsy.

**TABLE 3:** Association between eGDR and risk for poststroke adverse outcome.

Outcome	Case/Person‐years	Model 1		Model 2		Model 3	
		HR (95% CI)	*p*	HR (95% CI)	*p*	HR (95% CI)	*p*
**All poststroke adverse outcome cases**							
Q1 (< 6.525)	1114/39,025	Reference					
Q2 (6.525–8.494)	1181/39,492	1.04 (0.96–1.13)	0.290	1.05 (0.97–1.14)	0.252	1.05 (0.96–1.15)	0.250
Q3 (> 8.494)	429/17,877	0.82 (0.73–0.92)	< 0.001	0.82 (0.74–0.92)	< 0.001	0.83 (0.73–0.94)	0.004
**Poststroke depression**							
Q1 (< 6.525)	162/46,210	Reference					
Q2 (6.525–8.494)	179/46,473	1.07 (0.87–1.32)	0.527	1.06 (0.86–1.32)	0.566	1.07 (0.86–1.34)	0.523
Q3 (> 8.494)	50/20,461	0.64 (0.47–0.88)	0.006	0.64 (0.46–0.87)	0.005	0.66 (0.47–0.94)	0.022
**Poststroke disability**							
Q1 (< 6.525)	1038/41,539	Reference					
Q2 (6.525–8.494)	1068/41,650	1.02 (0.94–1.12)	0.622	1.03 (0.94–1.12)	0.555	1.02 (0.94–1.12)	0.592
Q3 (> 8.494)	398/18,757	0.83 (0.74–0.94)	0.002	0.84 (0.75–0.94)	0.003	0.84 (0.74–0.96)	0.011
**Poststroke epilepsy**							
Q1 (< 6.525)	154/46,522	Reference					
Q2 (6.525–8.494)	145/46,789	0.93 (0.74–1.16)	0.519	0.93 (0.74–1.16)	0.518	0.89 (0.70–1.13)	0.341
Q3 (> 8.494)	45/20,661	0.62 (0.44–0.87)	0.005	0.62 (0.44–0.86)	0.005	0.61 (0.42–0.89)	0.010
**Poststroke delirium**							
Q1 (< 6.525)	157/47,686	Reference					
Q2 (6.525–8.494)	166/47,795	1.04 (0.84–1.30)	0.699	1.07 (0.86–1.33)	0.542	1.08 (0.86–1.37)	0.494
Q3 (> 8.494)	34/21,078	0.53 (0.37–0.77)	< 0.001	0.55 (0.38–0.80)	0.002	0.57 (0.38–0.86)	0.007

*Note:* Model 1 adjusted for age, sex; Model 2 adjusted for age, sex, ethnicity, Townsend deprivation index (TDI), education; Model 3 adjusted for age, sex, ethnicity, TDI, education, smoking status, alcohol intake status, Body mass index (BMI), systolic blood pressure (SBP), diastolic blood pressure (DBP), sleep duration, metabolic syndrome (MetS) and healthy diet.

Abbreviations: CI, confidence interval; eGDR, estimated glucose disposal rate; HR, hazard ratio.

### 3.4 Sensitivity and Subgroup Analyses

To evaluate the robustness of our findings, we conducted multiple sensitivity analyses. These included additional adjustments for medication use and comorbidities, exclusion of participants with less than 5 years of follow‐up or those aged below 55 years, and removal of individuals who developed diabetes during follow‐up. All sensitivity analyses yielded results consistent with the primary findings. We further applied the Fine–Gray method to account for competing risks of death in both stroke events and poststroke adverse outcomes (Table S6–S11). Additionally, we analyzed the association between eGDR levels and poststroke adverse outcomes across different time windows (3 months, 6 months, and 1 year). Notably, higher eGDR levels demonstrated significant protective effects against multiple adverse outcomes, particularly poststroke delirium and disability, at the 1‐year time point (Table S12).

Subgroup analyses explored the influence of potential confounders on eGDR's associations with stroke and poststroke outcomes. While most stratified analyses showed no significant interactions (P interaction > 0.05), notable differences emerged across demographic characteristics. Compared to the lowest tertile of eGDR (< 6.525 mg/kg/min), subjects in the highest tertile (> 8.494 mg/kg/min) demonstrated substantially reduced stroke risk, with a 48% reduction in older participants and a 46% reduction in females, the finding was generally consistent with the estimates from nonlinear analyses based on sex‐specificity and age‐specificity (Figures S2–S4). Additionally, we observed that participants with lower BMI exhibited reduced stroke risk, with a significant interaction effect (P interaction = 0.027) (Table S13).

### 3.5 Mediation Analysis

**FIGURE 3:**
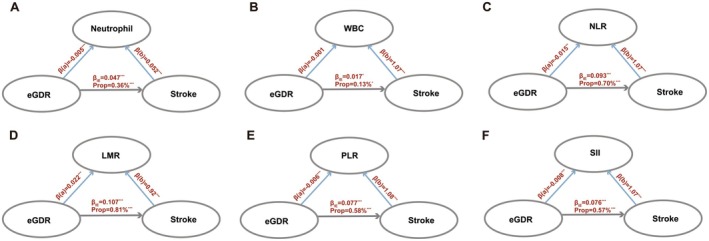
Mediation analysis of the relationship between eGDR and stroke through inflammatory markers. (A) Neutrophil; (B) WBC; (C) NLR; (D) LMR; (E) PLR; (F) SII. *Note:* Models adjusted for age, sex, ethnicity, education, Townsend deprivation index (TDI), smoking status, alcohol intake status, body mass index (BMI), systolic blood pressure (SBP), diastolic blood pressure (DBP), sleep duration, metabolic syndrome (MetS) and healthy diet. β_IE_ indicates the indirect effects. WBC, white blood cell; NLR, Neutrophil‐to‐lymphocyte ratio; LMR, lymphocytes‐to‐monocytes ratio; PLR, platelets‐to‐lymphocytes ratio; SII, systemic immune inflammation index.

After employing multivariate linear regression and Cox proportional hazards models with multiple testing corrections, we identified significant associations between specific inflammatory markers and both changes in eGDR and stroke risk. Most inflammatory markers exhibited significant differences between individuals in the lowest and highest eGDR tertiles, particularly in NLR (Cohen's d = 0.36, *p* < 0.001) (Figures S5 and S6 and Tables S14 and S15). Mediation analyses were performed to determine whether the relationship between eGDR and stroke risk is mediated through specific circulating biomarkers. After adjustment for confounders consistent with the primary analysis, significant partial mediation effects were observed for low‐grade inflammatory markers, including NLR, Neutrophil, WBC, LMR, PLR, and SII. These findings suggest that elevated insulin resistance may attenuate stroke risk through biological mechanisms involving modulation of inflammatory processes (Figure 3 and Table S16).

## 4 Discussion

In this prospective cohort study utilizing longitudinal follow‐up data from the UK Biobank, we found that elevated eGDR levels were significantly associated with decreased risk of stroke and poststroke adverse outcomes. Consistent findings were observed across both hemorrhagic and ischemic stroke subtypes. Notably, a high eGDR level was associated with a decreased risk of poor long‐term prognosis following stroke, independently predicting the risk of poststroke adverse outcomes including depression, disability, epilepsy, and delirium among stroke survivors. Furthermore, inflammatory markers partially mediated the association between eGDR and stroke, suggesting that low‐grade inflammation may represent a potential underlying mechanism through which impaired insulin sensitivity and resistance contribute to stroke occurrence.

Previously, Kernan and colleagues first identified insulin resistance as a potential stroke risk factor, linking it to metabolic, hematologic, and cellular events promoting atherosclerosis and thrombosis [34]. While the hyperinsulinemic‐euglycemic clamp remains the gold standard for assessing insulin resistance, its clinical application is limited by complexity and cost [35]. The eGDR, calculated from routine clinical parameters, has emerged as a practical alternative. Studies have shown eGDR's association with stroke risk and mortality in Type 2 diabetes patients, though underlying mechanisms remain unclear. The China National Stroke Registry III demonstrated eGDR's predictive value for 3‐month functional outcomes and 1‐year vascular events. However, the impact of eGDR on recognized poststroke adverse outcomes, including poststroke depression, disability, epilepsy, and delirium, remained unexplored in stroke survivors [16]. Our work builds upon previous studies by expanding several key aspects, encompassing a broader range of poststroke adverse outcomes and providing insights into potential inflammatory mechanisms.

In this study, over 13.9 years of follow‐up, we found that the highest eGDR tertile was associated with significantly reduced risks of developing stroke compared to the lowest tertile, with 45% for hemorrhagic stroke and 47% for ischemic stroke. Previous animal studies have simulated insulin resistance by activating insulin signaling pathways through acute insulin treatment of primary embryonic cortical neurons. This diabetes experimental model, involving various pretreatments of mouse cortical neurons, revealed exacerbated neuronal damage following ischemia–reperfusion injury under conditions of prolonged insulin resistance [36, 37]. Epidemiological studies have similarly demonstrated associations between insulin resistance and stroke risk. A prospective community‐based cohort study from Kailuan, encompassing 54,098 participants over 9 years of follow‐up, evaluated the relationship between cumulative insulin resistance surrogate marker triglyceride‐glucose index (TyG) and ischemic stroke, finding that elevated cumulative TyG increased the risk of ischemic stroke [38]. Alexander Zabala and colleagues analyzing data from the Swedish National Diabetes Registry between 2004 and 2016 observed reduced risks of all‐cause stroke and ischemic stroke with increasing eGDR levels, though no association was found with hemorrhagic stroke [39]. Additionally, a recent study from a Japanese multicenter study of 4655 acute ischemic stroke patients demonstrated a correlation between insulin resistance and poor functional outcomes using HOMA‐IR. However, most existing studies have relied on smaller sample sizes and cross‐sectional designs [40]. Our study extends previous research through a larger sample size, longer follow‐up, and comprehensive assessment of poststroke outcomes. We uniquely demonstrate that elevated eGDR levels are associated with reduced risks of poststroke adverse outcomes including depression, disability, epilepsy, and delirium.

Insulin resistance, manifesting through metabolic syndrome characteristics, affects over half of stroke patients. The multicenter randomized Insulin Resistance Intervention After Stroke (IRIS) trial demonstrated pioglitazone's effectiveness in secondary stroke prevention among prediabetic patients and recent post hoc analyses of the IRIS trial, which recruited participants with mild or no disability, focused on the risk of poststroke disability development [41]. Previous prospective multicenter cohort studies have also evaluated HOMA‐IR's potential predictive value in identifying poststroke depression [42]. Indeed, depression, disability, and cognitive decline, including epilepsy and delirium, are widely recognized as adverse poststroke outcomes [3]. Insulin resistance may participate in this complex pathogenesis, with microvascular dysfunction in prediabetes considered a key etiological factor, particularly given the not uncommon co‐occurrence of diabetes and stroke [43]. The mechanisms by which insulin resistance influences poststroke depression, disability, and cognitive impairment remain incompletely understood and complex. Current explanations encompass multiple levels of alterations, including changes in the hypothalamic–pituitary–adrenal axis and sympathetic nervous system, blood–brain barrier permeability modifications, and reduced cerebrovascular reactivity [44]. Poststroke stress response leads to hypothalamic–pituitary–adrenal axis hyperfunction, potentially causing neurotransmitter metabolic imbalance and altered neural circuit homeostasis, resulting in depressive states [45]. At the vascular level, insulin resistance tends to activate inflammatory factor release, compromising blood–brain barrier integrity and promoting neurotoxic substance infiltration into brain parenchyma. This, combined with endothelial dysfunction‐induced neurovascular uncoupling, collectively contributes to functional and structural alterations in small vessels, subsequently impairing brain tissue perfusion regulation [46]. Additionally, at the molecular level, insulin signaling pathway abnormalities affect neuronal energy metabolism, inducing mitochondrial dysfunction that exacerbates oxidative stress injury, reduces cerebrovascular reactivity, and impacts neuronal synaptic plasticity, accelerating cognitive decline [47]. While hemodynamic alterations likely drive the association between insulin resistance and poststroke outcomes, prestroke physiological and lifestyle factors may also contribute. Our findings establish eGDR as a promising clinical marker for predicting poststroke complications, though further validation studies are warranted.

Furthermore, this study provided additional sensitivity analysis evidence requiring adequate statistical power, rendering our findings independent of complex confounding factors. Multiple sensitivity analyses were conducted, considering not only the competing risk of death but also observing consistent associations between eGDR and the risk of stroke and adverse poststroke outcomes in nondiabetic individuals. Given that the duration of a disease can influence the risk of developing its associated complications, a segmented Cox model analysis revealed significant associations within the first year [48]. This suggests that the cumulative damage to the nervous system caused by insulin resistance and metabolic abnormalities is typically progressive. These findings underscore the importance of long‐term metabolic monitoring in stroke patients to mitigate the risk of adverse neurological outcomes. Intriguingly, subgroup analyses revealed stronger protective effects of high eGDR in elderly individuals (> 65 years) and females, highlighting the importance of early insulin resistance identification in these populations. The pronounced protective effect of elevated eGDR among older adults likely stems from age‐related metabolic changes. Previous research has demonstrated a significant association between insulin resistance and biological aging, with each unit increase in TyG index corresponding to a 1.64‐year increase in biological age and accelerated aging risk [49]. This suggests insulin resistance serves as a critical driver of biological aging, which may explain why maintaining lower insulin resistance (reflected by higher eGDR) confers more substantial stroke protection in elderly populations. Furthermore, a nationwide prospective cohort study from China revealed that persistently low eGDR significantly increased the risk of incident cardiovascular disease in middle‐aged and elderly participants [50]. This finding strongly aligns with our observation of enhanced eGDR protective effects in the elderly subgroup. Regarding sex differences, the stronger eGDR‐stroke risk association in females likely derives from several distinct physiological characteristics. Females typically exhibit higher cerebral blood flow velocities with greater vascular sensitivity to neurohumoral regulation, particularly from estrogen, making their cerebral vasculature more susceptible to mechanical stress under metabolic dysregulation [51, 52]. Furthermore, females generally engage less frequently in confounding lifestyle behaviors such as smoking and alcohol consumption, potentially allowing the protective effects of improved insulin sensitivity to manifest more prominently [53]. This observation aligns with previous research documenting more robust associations between insulin resistance and cardiovascular events in females [54].

Of note, previous studies have indicated that the association between insulin resistance and stroke outcomes might be mediated by elevated systemic inflammation. Numerous studies have confirmed a significant positive correlation between CRP and insulin resistance, supporting inflammation's independent association with both ischemic stroke risk and adverse prognosis [55]. Mechanistically, chronic insulin resistance affects glucose absorption and utilization, readily inducing oxidative stress and promoting inflammation, leading to platelet adhesion and aggregation that impair vascular endothelial cell function, accelerating thrombus formation and atherosclerotic plaque development and rupture [21]. A recent longitudinal study demonstrated that inflammatory markers mediate the association between the TyG and adverse cardiovascular events in hypertensive patients, suggesting the critical role of inflammatory mechanisms in insulin resistance‐mediated atherosclerosis [56]. Furthermore, evidence from both animal and human studies demonstrates that insulin resistance plays a pivotal role throughout the continuum of atherosclerotic plaque formation by potentiating chronic systemic inflammatory responses [57]. This influence permeates the complete pathological continuum, with the monocyte/macrophage axis participating in the progression from initial fatty streak development to advanced plaque formation [58]. Persistent low‐grade inflammation not only accelerates the late pathological manifestations of atherosclerosis but also induces vascular endothelial dysfunction. At the cellular level, perturbations in insulin signaling pathways further exacerbate endothelial dysfunction and promote foam cell formation. This pathological cascade highlights the interplay between insulin resistance, chronic inflammation, and endothelial dysfunction, which collectively drive the progression of atherosclerosis and its complications. Moreover, insulin resistance impairs the endothelium's vasoprotective and anti‐inflammatory functions, creating a vicious cycle that exacerbates vascular damage and increases susceptibility to stroke [21, 59]. This study incorporated multiple inflammatory markers for mediation analysis, providing preliminary evidence supporting the mechanistic link between eGDR and stroke risk. As expected, low‐grade inflammatory markers represented by NLR and SII demonstrated certain mediating effects.

Our study extends previous research by providing the first evaluation of eGDR's predictive role in stroke subtypes and poststroke adverse outcomes, including depression, disability, delirium, and epilepsy events, while elucidating the mediating effects of inflammatory markers in these associations. However, several limitations warrant consideration. Firstly, while eGDR serves as a practical surrogate measure of insulin resistance in large population studies, our study lacks validation against the hyperinsulinemic euglycemic clamp, which remains the gold standard for measuring insulin resistance. Secondly, UK Biobank participants are predominantly of European descent (> 90%), substantially limiting the generalizability of our findings. Given documented ethnic variations in stroke epidemiology and risk profiles, the predictive validity of eGDR may differ across populations, necessitating validation in ethnically diverse cohorts. Thirdly, despite adjusting for multiple covariates and employing sensitivity analyses, residual confounding remains a concern. Self‐reported data without clinical verification introduces potential recall bias, particularly for comorbidities and lifestyle factors affecting eGDR [60]. Additionally, unmeasured variables such as medication adherence, fluctuations in glycemic control, and weight variability, could alter eGDR values over time. Fourthly, this study is based on ICD‐10 codes for stroke classification and poststroke adverse outcome identification rather than neuroimaging data, limiting both diagnostic precision and anatomical localization capabilities [61, 62]. The specific location of stroke lesions (cortical versus deep structures, or involvement of frontal, parietal, and temporal lobes) significantly influences poststroke complications [63], yet remained unaccounted for in our analysis. Future research should incorporate radiological data to improve diagnostic accuracy and enable lesion‐specific outcome prediction models. Fifthly, a significant limitation is our inability to properly differentiate between new‐onset and recurrent stroke events, potentially impacting risk calculations. Although sensitivity analyses excluding participants with prior stroke history yielded consistent findings, future research should incorporate comprehensive pre‐enrollment records to accurately distinguish between initial and recurrent events. Finally, our study shares inherent limitations of observational cohort designs, including “healthy volunteer” selection bias and the inability to establish causality [64]. The baseline‐only assessment of eGDR further limits our ability to capture dynamic changes in insulin resistance over time. Future multicenter studies should evaluate multiple insulin resistance indicators, and their temporal relationships with stroke outcomes across diverse populations.

## 5 Conclusions

This study demonstrates that high eGDR levels were associated with reduced risk of stroke and poststroke adverse outcomes, providing preliminary evidence for understanding the inflammatory mechanisms underlying the relationship between insulin resistance and stroke. Our findings suggest that insulin resistance indicators, quantified by eGDR, may serve as promising therapeutic targets for both primary stroke prevention and secondary poststroke rehabilitation strategies, which have significant public health implications.

## Author Contributions

X.H., P.D., and Y.X. guided the literature review and planned the analyses. Y.L. and X.H. extracted the data from the UK Biobank database. Y.L., Z.B., and S.T. contributed to the acquisition and interpretation of data. J.L. and S.S. conceptualized the research aims. Y.X. and S.T. participated in data analysis and interpretation. X.H., P.D., and Y.X. wrote the first draft of the paper, and the other authors provided comments and approved the final manuscript. H.W. and J.L. read and approved the final manuscript. All authors read the manuscript and approved the final draft.

## Conflicts of Interest

The authors declare no conflicts of interest.

## Supporting information


Appendix S1.

## Data Availability

The data that support the findings of this study are available on request from the corresponding author. The data are not publicly available due to privacy or ethical restrictions.
