# Supplementary material for: Associations of Estimated Glucose Disposal Rate With Stroke Risk and Poststroke Adverse Outcomes: A Prospective Cohort Study
Source: CNS Neurosci Ther. 2025 Jun 5;31(6):e70420. doi: 10.1111/cns.70420 (PMC12141760; doi:10.1111/cns.70420)
Supplement: Supplementary file 1 — Appendix S1. [file CNS-31-e70420-s001.docx]

**Supplementary Materials**

[Supplementary Methods 3](#_Toc191931541)

[Supplementary Table S1. Definition of outcomes in the UK Biobank. 6](#_Toc191931542)

[Supplementary Table S2. Fields IDs of UK Biobank. 7](#_Toc191931543)

[Supplementary Table S3. The percentage of missing data in the present study. 8](#_Toc191931544)

[Supplementary Table S4. The variance inflation factors for all covariates. 9](#_Toc191931545)

[Supplementary Table S5. AIC/BIC in model selection 10](#_Toc191931546)

[Supplementary Table S6. Hazard ratios for eGDR associated with incident stroke in models further adjusted 11](#_Toc191931547)

[Supplementary Table S7. Hazard ratios for eGDR associated with incident stroke after excluding participants were **<** 55 years old at baseline 12](#_Toc191931548)

[Supplementary Table S8. Hazard ratios for eGDR associated with incident stroke after excluding participants with less than 5 years of follow-up 13](#_Toc191931549)

[Supplementary Table S9. Hazard ratios for eGDR associated with incident stroke after excluding participants with diabetes 14](#_Toc191931550)

[Supplementary Table S10. Hazard ratios for competitive risk analysis of eGDR associated with incident stroke outcomes 15](#_Toc191931551)

[Supplementary Table S11. Hazard ratios for competitive risk analysis of eGDR associated with poststroke adverse outcomes 16](#_Toc191931552)

[Supplementary Table S12. Hazard ratios for eGDR associated with poststroke adverse outcome at 3-month, 6-month, and 1-year follow-up 17](#_Toc191931553)

[Supplementary Table S13. Interaction terms for potential risk factors of eGDR associated with incident stroke and poststroke adverse outcomes 18](#_Toc191931554)

[Supplementary Table S14. Multivariable linear regression for the associations between eGDR and inflammation biomarkers. 19](#_Toc191931555)

[Supplementary Table S15. Longitudinal associations between the risk of incident stroke with inflammatory markers. 20](#_Toc191931556)

[Supplementary Table S16. The mediation effects of Inflammatory markers in the association between eGDR with stroke at baseline. 21](#_Toc191931557)

[Supplementary Figure S1. The directed acyclic graph (DAG) illustrates the relationship between the exposures (eGDR), the mediators, the outcome (Stroke), and the covariates included in the analyses. The direction of arrows in the acyclic graph was based on previous literature, The DAG was drawn using http://www.dagitty.net/. 22](#_Toc191931558)

[Supplementary Figure S2. Associations between eGDR and incidence of stroke, with separate analyses for sex and age. 23](#_Toc191931559)

[Supplementary Figure S3. Subgroup analysis of eGDR with incidence of stroke. 24](#_Toc191931560)

[Supplementary Figure S4. Subgroup analysis of eGDR with incidence of poststroke adverse outcomes. 25](#_Toc191931561)

[Supplementary Figure S5. Heatmap of associations between blood inflammatory markers with eGDR and incident stroke risks. 26](#_Toc191931562)

[Supplementary Figure S6. Differences in inflammatory markers between the lowest (Q1) and highest (Q3) eGDR tertiles. 27](#_Toc191931563)

# Supplementary Methods

1. **Covariates**

**1.1 Sociodemographic characteristics**

Age at baseline, sex, and the Townsend Deprivation Index (TDI), were obtained from local National Health Service (NHS) Primary Care Trust registries. Sex information was updated by participant response to 2 options from a questionnaire (female or male) if the participant reported this was incorrect. Hence this field may contain a mixture of the sex the NHS had recorded for the participant and self-reported sex. TDI was used as a measure of socioeconomic status (SED). This combines census data on housing, employment, social class, and car availability based on the postal code of participants. A greater TDI score implies a greater degree of deprivation and a low SED. Positive value of the index will indicate areas with high material deprivation, whereas that with negative values will indicate relative affluence. A score of 0 represents an area with overall mean values. SED was finally classified into high, medium, and low levels according to the TDI terciles in order from negative to positive ^[1]^.

**1.2 Sociodemographic information**

Ethnic background was classified as white and non-white. Education was classified as college or university degree, A levels/AS levels or equivalent, O levels/GCSEs or equivalent, CSEs or equivalent, NVQ or HND or HNC or equivalent and other professional qualifications, defined as lower or higher level.

**1.3 Lifestyle factors**

**1.3.1 Smoking status**

Participants were asked “Do you smoke tobacco now?” about current tobacco smoking with the multiple choices of answer as “Yes, on most or all days”, “Only occasionally”, and “No”. Participants were asked “In the past, how often have you smoked tobacco?” about current tobacco smoking with the multiple choices of answer as “Smoked on most or all days”, “Smoked occasionally”, “Just tried once or twice”, and “I have never smoked”. Smoking status summarizes the current/past smoking status of the participant and was classified as “Never” “Previous” and “Current”.

**1.3.2** **Alcohol intake status**

Participants were asked “About how often do you drink alcohol?” about alcohol intake frequency with the multiple choices of answer as “Daily or almost daily”, “Three or four times a week”, “Once or twice a week”, “One to three times a month”, “Special occasions only” and “Never”. Alcohol intake status summarizes the current/past smoking status of the participant and was classified as “Never” “Previous” and “Current”.

**1.3.3 Sleep duration**

Assessment of sleep traits contains data from the touchscreen questionnaire on duration of sleep, participants were asked “About how many hours sleep do you get in every 24 hours?” about the sleep duration on the touchscreen.

**1.3.4 Healthy diet and its components**

Healthy diet was based on consumption of more than 4 of the 7 routinely consumed kinds of food including^[2]^ 1) fruits ≥ 3 servings/day, 2) vegetables ≥ 3 servings/day, 3) fish ≥2 servings/week, 4) processed meats ≤ 1 serving/week,5) unprocessed red meats ≤ 1.5 servings/week, 6) whole grains ≥ 3servings/day, and 7) refined grains ≤ 1.5 servings/day, according to recommendations on dietary priorities, which were suggested to link to better cognition and dementia reduction.

- 1. **Information on comorbidities**

**1.4.1 Metabolic syndrome (MetS)**

MetS was defined using the harmonized definition when meeting 3 or more of the following metabolic criteria^[3]^: 1) waist circumference ≥102 cm in men and ≥88 cm in women, 2) serum triglycerides (TG) level ≥ 1.7 mmol/L, 3) high density lipoprotein (HDL) < 1.0 mmol/L in men and < 1.3 mmol/L in women, 4) systolic blood pressure (SBP) ≥130 mmHg and/or diastolic blood pressure (DBP) ≥ 85 mmHg, and 5) serum fasting glucose level ≥ 5.6 mmol/L.

**1.4.2 Physical frailty**

The Fried frailty phenotype used in the previous study was applied because it is based on physical-related frailty, including the following 5 criteria: weight loss, tiredness, low physical activity, slow walking speed, and low grip strength. Participants were defined as frail if they fulfilled 3 or more of the 5 criteria ^[4]^.

**1.4.3 Chronic diseases**

Chronic diseases were included the history of diabetes, hypercholesterolemia, hypertension, heart failure, and coronary heart disease were determined using primary and secondary diagnoses from the International Classification of Diseases, Tenth Revision (ICD-10), hospital admission data from England, Scotland, and Wales, and cancer registry data ^[5]^.

1. **Mediation analysis**

This study employed mediation analysis to evaluate the potential mediating role of blood biomarkers in the relationship between eGDR and stroke events. The mediation analysis was based on the causal inference framework proposed by Baron and Kenny, which requires three fundamental assumptions^[6]^: 1) a significant association between the independent variable (eGDR) and the dependent variable (stroke events); 2) a significant association between the independent variable and the hypothesized mediators (inflammatory biomarkers); and 3) a significant association between the mediators and the dependent variable while controlling for the independent variable.

A two-stage modeling approach was implemented for the mediation effect analysis. First, logistic regression models were used to assess the associations between eGDR and multiple inflammatory biomarkers (neutrophil count, lymphocyte count, monocyte count, platelet count, NLR, LMR, SII, and PLR). These models were adjusted for potential confounding factors including age, sex, ethnicity, TDI, education level, smoking status, alcohol consumption status, BMI, SBP, DBP, sleep duration, MetS, and healthy diet. Second, Cox proportional hazards regression models were employed to evaluate the associations between the mediators and stroke events, while simultaneously controlling for eGDR. This critical step ensures proper estimation of the direct effects by accounting for both the mediator and exposure in the same model.

Causal mediation analysis was performed using the "mediation" package ^[7]^ in R software with 1,000 nonparametric bootstrap resamples to estimate the total effect, direct effect (DE), and indirect effect (IDE)^[8]^. The total effect was decomposed into the direct effect (independent influence of eGDR on stroke events) and indirect effect (influence mediated through inflammatory biomarkers). The magnitude of mediation was quantified by calculating the proportion mediated.

The following was information for the regression equation in the single mediation analyses ^[9]^:

For the mediator model based on Logistic regression,

E[M|X, C] = α₀ + α₁X + α₂C

For the outcome model based on Cox regression,

E[Y|X, M, C] = β₀ + β₁X + β₂M + β₃C

where Y represented the outcome variable (stroke events), X represented the predictor variable (eGDR), M represented the mediator (inflammatory biomarkers including neutrophil count, lymphocyte count, monocyte count, platelet count, NLR, LMR, SII, and PLR), C represented the adjusting confounders including age, sex, ethnicity, TDI, education level, smoking status, alcohol consumption status, BMI, SBP, DBP, sleep duration, MetS, and healthy diet.

Using these models and the mediation package in R, we calculated the following causal effects ^[10]^:

1. Average Causal Mediation Effect (ACME) or Indirect Effect = α₁β₂
2. Average Direct Effect (ADE) = β₁
3. Total Effect = ADE + ACME = β₁ + α₁β₂

The proportion mediated was calculated as: proportion = ACME/Total Effect = α₁β₂ / (β₁ + α₁β₂)

All effect estimates were reported as hazard ratios (HRs) with 95% confidence intervals, and P-values were computed using the bootstrap method, the false discovery rate (FDR) was obtained using the Benjamini-Hochberg (BH) method to correct for multiple comparisons.

**Reference**

1. Zhang YB, Chen C, Pan XF, Guo J, Li Y, Franco OH, Liu G, Pan A. Associations of healthy lifestyle and socioeconomic status with mortality and incident cardiovascular disease: two prospective cohort studies. BMJ. 2021 Apr 14; 373: n604
2. Lourida I, Hannon E, Littlejohns TJ, et al. Association of Lifestyle and Genetic Risk With Incidence of Dementia. JAMA. 2019; 322(5): 430-437.
3. Machado-Fragua MD, Fayosse A, Yerramalla MS, et al. Association of Metabolic Syndrome With Incident Dementia: Role of Number and Age at Measurement of Components in a 28-Year Follow-up of the Whitehall II Cohort Study. Diabetes Care. 2022; 45(9): 2127-2135.
4. Petermann-Rocha F, Lyall DM, Gray SR, et al. Associations between physical frailty and dementia incidence: a prospective study from UK Biobank. Lancet Healthy Longev. 2020; 1(2): e58-e68.
5. Hsieh MT, Hsieh CY, Tsai TT, et al. Performance of ICD-10-CM Diagnosis Codes for Identifying Acute Ischemic Stroke in a National Health Insurance Claims Database. Clin Epidemiol. 2020 Sep 25; 12: 1007-1013.
6. Feng J, Wu Y, Meng M, et al. The mediating effect of blood biomarkers in the associations between inflammatory bowel disease and incident psychiatric disorders: a prospective cohort study. Int J Surg. 2024 Dec 1;110(12):7738-7748.
7. Liao H, Cheng J, Pan D, et al. Association of earlier age at menopause with risk of incident dementia, brain structural indices and the potential mediators: a prospective community-based cohort study. EClinicalMedicine. 2023 Jun 8; 60:102033.
8. Sankaran K, Holmes S. structSSI: Simultaneous and Selective Inference for Grouped or Hierarchically Structured Data. J Stat Softw. 2014; 59(13): 1-21
9. Baron RM, Kenny DA. The moderator-mediator variable distinction in social psychological research: conceptual, strategic, and statistical considerations. J Pers Soc Psychol. 1986; 51(6): 1173-1182.
10. Pati D. Improving Precision and Confidence of Research Application Through Mediator and Moderator Models. HERD. 2020 Apr;13(2):12-20.

# Supplementary Table S1. Definition of outcomes in the UK Biobank.

| **Outcome** | **ICD10 code** |
| --- | --- |
| Stroke incident | |
| Stroke | H341, I63, I693, G46, I60, I61, I62, I690, I691, I692, I64, I694 |
| Ischemic Stroke | I60.0, I60.1, I60.2, I61.1, I61.2, I61.3, I61.4, I61.5, I61.6 |
| Hemorrhagic Stroke | I63.0, I63.1, I63.2, I63.4, I63.5, I63.6 |
| Poststroke adverse outcomes | |
| Depression | F32, F33, F34, F38, F39 |
| Disability | Z736, Z74, F40–F45, F50, F52, F54–F55, F59, F80, H53-54, H90–H91, R40–R49, R53–R54 |
| Delirium | F05 |
| Epilepsy | G40 |

# Supplementary Table S2. Fields IDs of UK Biobank.

| **Fields** | **Variable** |
| --- | --- |
| eGDR | |
| 48 | Waist |
| 30751 | HbA1c |
| 20002/6150/41270 | Hypertension |
| 20003/6177/6153 | Mediation |
| 4080 | Systolic blood pressure |
| 4090 | Diastolic blood pressure |
| Confounding Variables | |
| 31 | Sex |
| 22189 | Townsend deprivation index at recruitment |
| 6138 | Education |
| 21003 | Age when attended assessment centre |
| 21001 | Body mass index (BMI) |
| 21000 | Ethnic background |
| 20117 | Alcohol intake status |
| 20116 | Smoking Status |
| 6177/6153 | Mediation |
| 1070/1080/1090 | Total sedentary behavior |
| 1160 | Sleep duration |
| Comorbidities | |
| 20002/1220/1222/1223/130708/130710 130712/6177/6153/41280 | Diabetes |
| 20002/41270/41280 | Coronary heart disease |
| 20002/41270 | Hyperlipidemia |
| 130814 | Heart failure |
| 20002/41270/41280 | Stroke |
| 1309/1319/1289/1299/1369/1379/1389/1349  /1329/6144  2306/ 2080/22037/22038/ 22039/ 924/ 46/ 47  48/30760/30870/30740/4080/4090 | Health diet  Physical frailty  MetS |
| Inflammation markers | |
| 30000 | White blood cell (leukocyte) count |
| 30080 | Platelet count |
| 30120 | Lymphocyte count |
| 30130 | Monocyte count |
| 30140 | Neutrophil count |
| 30180 | Lymphocyte percentage |
| 30190 | Monocyte percentage |
| 30200 | Neutrophil percentage |
| 30710 | C-reactive protein |

# Supplementary Table S3. The percentage of missing data in the present study.

| **Characteristics** | **eGDR(mg/kg/min)** | | |
| --- | --- | --- | --- |
|  | **Q1 (<6.525)** | **Q1 (<6.525)** | **Q1 (<6.525)** |
| Age, years | 154,185 (100%) | 154,181 (100%) | 154,183 (100%) |
| Sex | 154,185 (100%) | 154,181 (100%) | 154,183 (100%) |
| TDI | 153,988 (99.9%) | 154,010 (99.9%) | 153,977 (99.9%) |
| Missing | 197 (0.1%) | 171 (0.1%) | 206 (0.1%) |
| Ethnicity | 153,641 (99.7%) | 153,541 (99.6%) | 153,649 (99.7%) |
| Missing | 544 (0.3%) | 640 (0.4%) | 534 (0.3%) |
| Education | 121,990 (79.2%) | 122,120 (79.3%) | 131,353 (86.2%) |
| Missing | 32,195 (20.8%) | 32,061 (20.7%) | 22,830 (14.8%) |
| Smoking status | 153549 (99.6%) | 153,494 (99.6%) | 153,636 (99.7%) |
| Missing | 636 (0.4%) | 687 (0.4%) | 547 (0.3%) |
| Alcohol intake status | 153,954 (99.9%) | 153,931 (99.9%) | 154,120 (99.7%) |
| Missing | 231 (0.1%) | 250 (0.1%) | 63 (0.3%) |
| Healthy diet | 118,910 (77.2%) | 119,429 (77.5%) | 120,668 (79.3%) |
| Missing | 35,275 (22.8%) | 34,752 (22.5%) | 33,515 (21.7%) |
| BMI, kg/m² | 153,226 (99.4%) | 153,218 (99.4%) | 153,459 (99.6%) |
| Missing | 959 (0.6%) | 963 (0.6%) | 724 (0.4%) |
| Waist circumference, cm | 154,185 (100%) | 154,181 (100%) | 154,183 (100%) |
| DBP, mmHg | 148,763 (96.5%) | 146,749 (95.2%) | 137,584 (89.3%) |
| Missing | 5,422 (3.5%) | 7,432 (4.8%) | 16,599 (10.7%) |
| SBP, mmHg | 148,765 (96.5%) | 146,753 (95.2%) | 137,589 (89.3%) |
| Missing | 5,420 (3.5%) | 7,428 (4.8%) | 16,594 (10.7%) |
| Sleep duration | 153,086 (99.3%) | 153,047 (99.3%) | 153,389 (99.5%) |
| Missing | 1,099 (0.7%) | 1,134 (0.7%) | 794 (0.5%) |
| Total physical activity | 119,548 (78.6%) | 119,608 (78.6%) | 123,629 (81.2%) |
| Missing | 34,637 (22.4%) | 34,573 (22.4%) | 30,554 (19.8%) |
| MetS | 154,185 (100%) | 154,181 (100%) | 154,183 (100%) |
| Coronary heart disease | 154,185 (100%) | 154,181 (100%) | 154,183 (100%) |
| Hyperlipidemia | 154,185 (100%) | 154,181 (100%) | 154,183 (100%) |
| Physical frailty | 154,185 (100%) | 154,181 (100%) | 154,183 (100%) |
| Diabetes | 154,185 (100%) | 154,181 (100%) | 154,183 (100%) |
| Hypertension | 154,185 (100%) | 154,181 (100%) | 154,183 (100%) |
| Stroke | 154,185 (100%) | 154,181 (100%) | 154,183 (100%) |
| Diabetes | 154,185 (100%) | 154,181 (100%) | 154,183 (100%) |
| Hypertension | 154,185 (100%) | 154,181 (100%) | 154,183 (100%) |

Note: BMI, body mass index, TDI, Townsend deprivation index, eGDR, Estimated glucose disposal rate, HDL, high-density lipoprotein cholesterol. SBP, systolic blood pressure, DBP, diastolic blood pressure.

# Supplementary Table S4. The variance inflation factors for all covariates.

| **Variable** | **VIF** |
| --- | --- |
| eGDR | 1.449442 |
| Sex | 1.047144 |
| Age | 1.056923 |
| TDI | 1.436011 |
| Ethnicity | 1.045222 |
| Education | 1.021034 |
| Smoling status | 2.290716 |
| Alcohol drinker status | 1.016784 |
| SBP | 1.793504 |
| DBP | 1.853015 |
| BMI | 1.100094 |
| MetS | 1.274719 |
| Health diet | 1.015510 |
| Sleep duration | 1.010853 |

Note: BMI, body mass index, TDI, Townsend deprivation index, eGDR, Estimated glucose disposal rate, SBP, systolic blood pressure, DBP, diastolic blood pressure.

# Supplementary Table S5. AIC/BIC in model selection

| **Criterion** | **Model 1** | **Model 2** | **Model 3** |
| --- | --- | --- | --- |
| AIC | 314011.5 | 296307.8 | 235815.9 |
| BIC | 314033.8 | 296359.8 | 235897.0 |

Note: Model 1 adjusted for age, sex; Model 2 adjusted for age, sex, ethnicity, Townsend deprivation index (TDI), education; Model 3 adjusted for age, sex, ethnicity, TDI, education, smoking status, alcohol intake status, Body mass index (BMI), systolic blood pressure (SBP), diastolic blood pressure (DBP), sleep duration, metabolic syndrome (MetS) and healthy diet. eGDR, Estimated glucose disposal rate; HR, Hazard ratio; CI, Confidence interval.

Abbreviations: eGDR, Estimated glucose disposal rate; AIC, Akaike Information Criterion; BIC, Bayesian Information Criterion; HR, Hazard ratio; CI, Confidence interval.

# Supplementary Table S6. Hazard ratios for eGDR associated with incident stroke in models further adjusted

| **Outcome** | **Case/Person-years** | **Model 4** | | **Model 5** | | **Model 6** | |
| --- | --- | --- | --- | --- | --- | --- | --- |
|  |  | **HR (95%CI)** | ***P*** | **HR (95%CI)** | ***P*** | **HR (95%CI)** | ***P*** |
| **Stroke** | | | | | | | |
| eGDR | 12,325/6,363,081 | 0.91 (0.90-0.92) | <0.001 | 0.96 (0.95-0.97) | <0.001 | 0.97 (0.96-0.98) | <0.001 |
| Q1 (<6.525) | 5,056/2,114,800 | Reference | | | | | |
| Q2 (6.525-8.494) | 5,107/2,114,874 | 1.03 (0.99-1.07) | 0.129 | 1.02 (0.98-1.07) | 0.251 | 1.02 (0.98-1.06) | 0.389 |
| Q3 (>8.494) | 2,162/2,133,407 | 0.56 (0.53-0.60) | <0.001 | 0.78 (0.73-0.82) | <0.001 | 0.80 (0.76-0.85) | <0.001 |
| **Hemorrhagic stroke** | | | | | | | |
| eGDR | 3,206/6,363,554 | 0.92 (0.90-0.93) | <0.001 | 0.97 (0.95-0.99) | 0.008 | 0.97 (0.95-0.99) | 0.018 |
| Q1 (<6.525) | 1,262/2,114,958 | Reference | | | | | |
| Q2 (6.525-8.494) | 1,314/2,115,102 | 1.03 (0.95-1.12) | 0.402 | 1.02 (0.94-1.11) | 0.620 | 1.02 (0.94-1.10) | 0.656 |
| Q3 (>8.494) | 630/2,133,493 | 0.57 (0.51-0.64) | <0.001 | 0.80 (0.71-0.90) | <0.001 | 0.82 (0.73-0.92) | <0.001 |
| **Ischemic stroke** | | | | | | | |
| eGDR | 8,781/6,363,634 | 0.91(0.90-0.92) | <0.001 | 0.96 (0.95-0.97) | <0.001 | 0.97 (0.95-0.98) | <0.001 |
| Q1 (<6.525) | 3,633/2,115,045 | Reference | | | | | |
| Q2 (6.525-8.494) | 3,659/2,115,113 | 1.04 (0.99-1.09) | 0.108 | 1.03 (0.98-1.08) | 0.224 | 1.02 (0.97-1.07) | 0.332 |
| Q3 (>8.494) | 1,489/2,133,477 | 0.57 (0.53-0.61) | <0.001 | 0.79 (0.73-0.85) | <0.001 | 0.82 (0.76-0.88) | <0.001 |

Note: Model 4 adjusted for age, sex, ethnicity, Townsend deprivation index (TDI), education, smoking status, alcohol drink status, Body mass index (BMI), systolic blood pressure (SBP), diastolic blood pressure (DBP), sleep duration, metabolic syndrome (MetS), healthy diet, lipid lowering medications, aspirin, antihypertensive drugs. Model 5 adjusted for age, sex, ethnicity, TDI, education, smoking status, alcohol drink status, BMI, SBP, DBP, sleep duration, MetS, healthy diet, frailty, Coronary Heart Disease (CHD), Heart Failure (HF), diabetes, hyperlipidemia and hypertension; Model 6 adjusted for age, sex, ethnicity, TDI, education, smoking status, alcohol drink status, BMI, SBP, DBP, sleep duration, MetS, healthy diet, lipid lowering medications, aspirin, antihypertensive drugs, frailty, CHD, HF, diabetes, hyperlipidemia and hypertension.

Abbreviations: eGDR, Estimated glucose disposal rate; HR, Hazard ratio; CI, Confidence interval.

# Supplementary Table S7. Hazard ratios for eGDR associated with incident stroke after excluding participants were < 55 years old at baseline

| **Outcome** | **Case/Person-years** | **Model 1** | | **Model 2** | | **Model 3** | |
| --- | --- | --- | --- | --- | --- | --- | --- |
|  |  | **HR (95%CI)** | ***P*** | **HR (95%CI)** | ***P*** | **HR (95%CI)** | ***P*** |
| **Stroke** | | | | | | | |
| eGDR | 10,267/3,879,304 | 0.90 (0.89-0.90) | <0.001 | 0.90 (0.89-0.91) | <0.001 | 0.91(0.89-0.92) | <0.001 |
| Q1 (<6.525) | 4,265/96,600 | Reference | | | | | |
| Q2 (6.525-8.494) | 4,332/97,243 | 1.00 (0.96-1.05) | 0.878 | 1.02 (0.97-1.06) | 0.419 | 1.04 (0.99-1.08) | 0.116 |
| Q3 (>8.494) | 474/1,116,919 | 0.52 (0.49-0.55) | <0.001 | 0.54 (0.51-0.57) | <0.001 | 0.54 (0.51-0.58) | <0.001 |
| **Hemorrhagic stroke** | | | | | | | |
| eGDR | 2,589/3,879,720 | 0.92 (0.90-0.94) | <0.001 | 0.92 (0.91-0.94) | <0.001 | 0.91 (0.89-0.93) | 0.018 |
| Q1 (<6.525) | 3,113/97,752 | Reference | | | | | |
| Q2 (6.525-8.494) | 3,148/98,427 | 1.03 (0.94-1.12) | 0.536 | 1.04 (0.95-1.13) | 0.620 | 1.02 (0.94-1.12) | 0.586 |
| Q3 (>8.494) | 1,180/116,981 | 0.60 (0.54-0.67) | <0.001 | 0.62 (0.55-0.69) | <0.001 | 0.58 (0.51-0.65) | <0.001 |
| **Ischemic stroke** | | | | | | | |
| eGDR | 7441/3,879,795 | 0.89(0.88-0.90) | <0.001 | 0.90 (0.89-0.91) | <0.001 | 0.90 (0.89-0.92) | <0.001 |
| Q1 (<6.525) | 1,037/99,828 | Reference | | | | | |
| Q2 (6.525-8.494) | 1,078/100,497 | 1.00 (0.95-1.05) | 0.955 | 1.01 (0.96-1.06) | 0.572 | 1.04 (0.99-1.10) | 0.108 |
| Q3 (>8.494) | 474/116,996 | 0.57 (0.53-0.61) | <0.001 | 0.53 (0.49-0.57) | <0.001 | 0.54 (0.50-0.59) | <0.001 |

Note: Model 1 adjusted for age, sex; Model 2 adjusted for age, sex, ethnicity, Townsend deprivation index (TDI), education; Model 3 adjusted for age, sex, ethnicity, TDI, education, smoking status, alcohol intake status, Body mass index (BMI), systolic blood pressure (SBP), diastolic blood pressure (DBP), sleep duration, metabolic syndrome (MetS) and healthy diet.

Abbreviations: eGDR, Estimated glucose disposal rate; HR, Hazard ratio; CI, Confidence interval.

# Supplementary Table S8. Hazard ratios for eGDR associated with incident stroke after excluding participants with less than 5 years of follow-up

| **Outcome** | **Case/Person-years** | **Model 1** | | **Model 2** | | **Model 3** | |
| --- | --- | --- | --- | --- | --- | --- | --- |
|  |  | **HR (95%CI)** | ***P*** | **HR (95%CI)** | ***P*** | **HR (95%CI)** | ***P*** |
| **Stroke** | | | | | | | |
| eGDR | 9,297/6,354,760 | 0.89 (0.88-0.90) | <0.001 | 0.90 (0.89-0.91) | <0.001 | 0.90 (0.89-0.91) | <0.001 |
| Q1 (<6.525) | 3,828/149,129 | Reference | | | | | |
| Q2 (6.525-8.494) | 3,822/2,111,402 | 0.98 (0.95-1.03) | 0.657 | 1.00 (0.99-1.05) | 0.904 | 1.02 (0.98-1.07) | 0.362 |
| Q3 (>8.494) | 1,647/2,132,032 | 0.51 (0.48-0.54) | <0.001 | 0.53 (0.50-0.56) | <0.001 | 0.53 (0.49-0.56) | <0.001 |
| **Hemorrhagic stroke** | | | | | | | |
| eGDR | 2,374/63,554,973 | 0.91 (0.89-0.92) | <0.001 | 0.91 (0.89-0.93) | <0.001 | 0.90 (0.88-0.92) | <0.001 |
| Q1 (<6.525) | 2,765/150,192 | Reference | | | | | |
| Q2 (6.525-8.494) | 2,769/2,111,495 | 1.03 (0.94-1.13) | 0.482 | 1.04 (0.95-1.14) | 0.385 | 1.03 (0.94-1.13) | 0.507 |
| Q3 (>8.494) | 1,155/2,132,052 | 0.56 (0.50-0.63) | <0.001 | 0.58 (0.52-0.65) | <0.001 | 0.54 (0.47-0.61) | <0.001 |
| **Ischemic stroke** | | | | | | | |
| eGDR | 6,689/635,469 | 0.89 (0.88-0.90) | <0.001 | 0.89 (0.88-0.91) | <0.001 | 0.90 (0.89-0.91) | <0.001 |
| Q1 (<6.525) | 939/152,018 | Reference | | | | | |
| Q2 (6.525-8.494) | 977/2,111,512 | 1.00 (0.95-1.05) | 0.955 | 1.01 (0.96-1.06) | 0.572 | 1.03 (0.98-1.09) | 0.108 |
| Q3 (>8.494) | 458/2,132,056 | 0.57 (0.53-0.61) | <0.001 | 0.53 (0.49-0.57) | <0.001 | 0.53 (0.49-0.58) | <0.001 |

Note: Model 1 adjusted for age, sex; Model 2 adjusted for age, sex, ethnicity, Townsend deprivation index (TDI), education; Model 3 adjusted for age, sex, ethnicity, TDI, education, smoking status, alcohol intake status, Body mass index (BMI), systolic blood pressure (SBP), diastolic blood pressure (DBP), sleep duration, metabolic syndrome (MetS) and healthy diet.

Abbreviations: eGDR, Estimated glucose disposal rate; HR, Hazard ratio; CI, Confidence interval.

# Supplementary Table S9. Hazard ratios for eGDR associated with incident stroke after excluding participants with diabetes

| **Outcome** | **Case/Person-years** | **Model 1** | | **Model 2** | | **Model 3** | |
| --- | --- | --- | --- | --- | --- | --- | --- |
|  |  | **HR (95%CI)** | ***P*** | **HR (95%CI)** | ***P*** | **HR (95%CI)** | ***P*** |
| **Stroke** | | | | | | | |
| eGDR | 9,297/6,354,760 | 0.89 (0.88-0.90) | <0.001 | 0.9 (0.89-0.91) | <0.001 | 0.89 (0.88-0.90) | <0.001 |
| Q1 (<6.525) | 3,824/1,746,767 | Reference | | | | | |
| Q2 (6.525-8.494) | 4,242/1,891,054 | 1.01 (0.97-1.06) | 0.577 | 1.03 (0.98-1.07) | 0.245 | 1.03 (0.98-1.08) | 0.202 |
| Q3 (>8.494) | 1,958/2,036,278 | 0.52 (0.49-0.55) | <0.001 | 0.53 (0.50-0.56) | <0.001 | 0.52 (0.49-0.55) | <0.001 |
| **Hemorrhagic stroke** | | | | | | | |
| eGDR | 2,374/63,554,973 | 0.91 (0.89-0.92) | <0.001 | 0.91 (0.89-0.93) | <0.001 | 0.90 (0.88-0.92) | <0.001 |
| Q1 (<6.525) | 2,701/1,746,967 | Reference | | | | | |
| Q2 (6.525-8.494) | 2,992/1,891,234 | 1.05 (0.96-1.14) | 0.294 | 1.05 (0.97-1.15) | 0.218 | 1.04 (0.95-1.14) | 0.356 |
| Q3 (>8.494) | 1,336/2,036,347 | 0.58 (0.52-0.64) | <0.001 | 0.59 (0.53-0.65) | <0.001 | 0.54 (0.47-0.61) | <0.001 |
| **Ischemic stroke** | | | | | | | |
| eGDR | 6,689/635,469 | 0.88 (0.87-0.89) | <0.001 | 0.89 (0.88-0.90) | <0.001 | 0.89 (0.87-0.90) | <0.001 |
| Q1 (<6.525) | 1,009/1,746,872 | Reference | | | | | |
| Q2 (6.525-8.494) | 1,155/1,891,255 | 1.01 (0.96-1.06) | 0.703 | 1.03 (0.97-1.08) | 0.572 | 1.04 (0.98-1.09) | 0.193 |
| Q3 (>8.494) | 583/2,036,352 | 0.51 (0.48-0.55) | <0.001 | 0.54 (0.50-0.57) | <0.001 | 0.52 (0.48-0.56) | <0.001 |

Note: Model 1 adjusted for age, sex; Model 2 adjusted for age, sex, ethnicity, Townsend deprivation index (TDI), education; Model 3 adjusted for age, sex, ethnicity, TDI, education, smoking status, alcohol intake status, Body mass index (BMI), systolic blood pressure (SBP), diastolic blood pressure (DBP), sleep duration, metabolic syndrome (MetS) and healthy diet.

Abbreviations: eGDR, Estimated glucose disposal rate; HR, Hazard ratio; CI, Confidence interval.

# Supplementary Table S10. Hazard ratios for competitive risk analysis of eGDR associated with incident stroke outcomes

| **Outcome** | **Case/Person-years** | **Model 1** | | **Model 2** | | **Model 3** | |
| --- | --- | --- | --- | --- | --- | --- | --- |
|  |  | **HR (95%CI)** | ***P*** | **HR (95%CI)** | ***P*** | **HR (95%CI)** | ***P*** |
| **Stroke** | | | | | | | |
| eGDR | 9,297/6,354,760 | 0.89 (0.88-0.90) | <0.001 | 0.90 (0.89-0.91) | <0.001 | 0.90 (0.88-0.91) | <0.001 |
| Q1 (<6.525) | 3,824/1,746,767 | Reference | | | | | |
| Q2 (6.525-8.494) | 4,242/1,891,054 | 1.00 (0.95-1.05) | 0.900 | 1.01 (0.96-1.06) | 0.730 | 1.03 (0.98-1.08) | 0.202 |
| Q3 (>8.494) | 1,958/2,036,278 | 0.50 (0.47-0.53) | <0.001 | 0.52 (0.49-0.55) | <0.001 | 0.52 (0.49-0.55) | <0.001 |
| **Hemorrhagic stroke** | | | | | | | |
| eGDR | 2,374/63,554,973 | 0.91 (0.89-0.92) | <0.001 | 0.91 (0.89-0.93) | <0.001 | 0.89 (0.87-0.92) | <0.001 |
| Q1 (<6.525) | 2,701/1,746,967 | Reference | | | | | |
| Q2 (6.525-8.494) | 2,992/1,891,234 | 1.00 (0.90-1.11) | 0.970 | 1.00 (0.91-1.11) | 0.940 | 0.98 (0.88-1.10) | 0.770 |
| Q3 (>8.494) | 1,336/2,036,347 | 0.53 (0.47-0.61) | <0.001 | 0.54 (0.48-0.62) | <0.001 | 0.48 (0.42-0.57) | <0.001 |
| **Ischemic stroke** | | | | | | | |
| eGDR | 6,689/635,469 | 0.89 (0.88-0.90) | <0.001 | 0.89 (0.88-0.91) | <0.001 | 0.90 (0.89-0.91) | <0.001 |
| Q1 (<6.525) | 1,009/1,746,872 | Reference | | | | | |
| Q2 (6.525-8.494) | 1,155/1,891,255 | 1.00 (0.94-1.05) | 0.870 | 1.01 (0.96-1.07) | 0.750 | 1.04 (0.98-1.09) | 0.193 |
| Q3 (>8.494) | 583/2,036,352 | 0.50 (0.46-0.53) | <0.001 | 0.52 (0.49-0.56) | <0.001 | 0.52 (0.48-0.56) | <0.001 |

Note: Model 1 adjusted for age, sex; Model 2 adjusted for age, sex, ethnicity, Townsend deprivation index (TDI), education; Model 3 adjusted for age, sex, ethnicity, TDI, education, smoking status, alcohol intake status, Body mass index (BMI), systolic blood pressure (SBP), diastolic blood pressure (DBP), sleep duration, metabolic syndrome (MetS) and healthy diet.

Abbreviations: eGDR, Estimated glucose disposal rate; HR, Hazard ratio; CI, Confidence interval.

# Supplementary Table S11. Hazard ratios for competitive risk analysis of eGDR associated with poststroke adverse outcomes

| **Outcome** | **Case/Person-years** | **Model 1** | | **Model 2** | | **Model 3** | |
| --- | --- | --- | --- | --- | --- | --- | --- |
|  |  | **HR (95%CI)** | ***P*** | **HR (95%CI)** | ***P*** | **HR (95%CI)** | ***P*** |
| **All poststroke adverse outcome cases** | | | | | | | |
| Q1 (<6.525) | 1,114/39,025 | Reference | | | | | |
| Q2 (6.525-8.494) | 1,181/39,492 | 1.02 (0.4-1.10) | 0.64 | 1.02 (0.94-1.11) | 0.63 | 1.01 (0.92-1.09) | 0.88 |
| Q3 (>8.494) | 429/17,877 | 0.81(0.73-0.91) | <0.001 | 0.81 (0.720.90) | <0.001 | 0.78 (0.69-0.89) | <0.001 |
| **Poststroke depression** | | | | | | | |
| Q1 (<6.525) | 1,262/2,114,958 | Reference | | | | | |
| Q2 (6.525-8.494) | 1,314/2,115,102 | 1.10 (0.89-1.37) | 0.360 | 1.10 (0.89-1.36) | 0.400 | 1.08 (0.86-1.35) | 0.490 |
| Q3 (>8.494) | 630/2,133,493 | 0.66 (0.48-0.91) | 0.011 | 0.65 (0.48-0.90) | 0.008 | 0.85 (0.75-0.96) | 0.010 |
| **Poststroke disability** | | | | | | | |
| Q1 (<6.525) | 3,633/2,115,045 | Reference | | | | | |
| Q2 (6.525-8.494) | 3,659/2,115,113 | 1.03 (0.95-1.12) | 0.497 | 1.03 (0.94-1.12) | 0.560 | 1.03 (0.94-1.13) | 0.464 |
| Q3 (>8.494) | 1489/2,133,477 | 0.84 (0.75-0.95) | 0.004 | 0.87 (0.77-0.97) | 0.016 | 0.83 (0.73-0.95) | 0.006 |
| **Poststroke epilepsy** | | | | | | | |
| Q1 (<6.525) | 3,633/2,115,045 | Reference | | | | | |
| Q2 (6.525-8.494) | 3,659/2,115,113 | 0.94 (0.75-1.17) | 0.570 | 0.94 (0.75-1.17) | 0.560 | 0.89 (0.70-1.13) | 0.330 |
| Q3 (>8.494) | 1,489/2,133,477 | 0.63 (0.45-0.88) | 0.006 | 0.62 (0.45-0.87) | 0.006 | 0.59 (0.41-0.86) | 0.006 |
| **Poststroke delirium** | | | | | | | |
| Q1 (<6.525) | 3,633/2,115,045 | Reference | | | | | |
| Q2 (6.525-8.494) | 3,659/2,115,113 | 1.04 (0.83-1.29) | 0.740 | 1.05 (0.84-1.31) | 0.660 | 1.06 (0.84-1.34) | 0.620 |
| Q3 (>8.494) | 1,489/2,133,477 | 0.54 (0.37-0.78) | 0.001 | 0.55 (0.38-0.80) | 0.002 | 0.53 (0.50-0.57) | <0.001 |

Note: Model 1 adjusted for age, sex; Model 2 adjusted for age, sex, ethnicity, Townsend deprivation index (TDI), education; Model 3 adjusted for age, sex, ethnicity, TDI, education, smoking status, alcohol intake status, Body mass index (BMI), systolic blood pressure (SBP), diastolic blood pressure (DBP), sleep duration, metabolic syndrome (MetS) and healthy diet.

Abbreviations: eGDR, Estimated glucose disposal rate; HR, Hazard ratio; CI, Confidence interval.

# Supplementary Table S12. Hazard ratios for eGDR associated with poststroke adverse outcome at 3-month, 6-month, and 1-year follow-up

| **Outcome** | **Model** | **Q1 (<6.525)** | | **Q2 (6.525-8.494)** | | **Q3 (>8.494)** | |
| --- | --- | --- | --- | --- | --- | --- | --- |
|  |  | **HR (95%CI)** | ***P*** | **HR (95%CI)** | ***P*** | **HR (95%CI)** | ***P*** |
| **3 months** | | | | | | | |
| Stroke→Depression | Unadjusted | Ref | Ref | 0.94 (0.54-1.65) | 0.837 | 0.62 (0.27-1.43) | 0.262 |
|  | Adjusted | Ref | Ref | 0.99 (0.55-1.78) | 0.969 | 0.59 (0.23-1.51) | 0.274 |
| Stroke→Disability | Unadjusted | Ref | Ref | 1.06 (0.95-1.19) | 0.293 | 0.95 (0.81-1.10) | 0.481 |
|  | Adjusted | Ref | Ref | 1.08 (0.96-1.22) | 0.186 | 0.97 (0.81-1.15) | 0.686 |
| Stroke→Epilepsy | Unadjusted | Ref | Ref | 2.40 (0.99-5.78) | 0.051 | 0.96 (0.25-3.70) | 0.949 |
|  | Adjusted | Ref | Ref | 2.31 (0.92-5.85) | 0.076 | 0.96 (0.22-4.20) | 0.958 |
| Stroke→Delirium | Unadjusted | Ref | Ref | 1.29 (0.74-2.25) | 0.365 | 0.50 (0.19-1.33) | 0.165 |
|  | Adjusted | Ref | Ref | 1.43 (0.79-2.57) | 0.235 | 0.66 (0.23-1.90) | 0.44 |
| **6 months** | | | | | | | |
| Stroke→Depression | Unadjusted | Ref | Ref | 1.12 (0.68-1.84) | 0.66 | 0.92 (0.47-1.79) | 0.796 |
|  | Adjusted | Ref | Ref | 1.16 (0.69-1.96) | 0.583 | 0.93 (0.43-2.00) | 0.851 |
| Stroke→Disability | Unadjusted | Ref | Ref | 1.06 (0.95-1.18) | 0.325 | 0.92 (0.79-1.07) | 0.277 |
|  | Adjusted | Ref | Ref | 1.08 (0.96-1.22) | 0.19 | 0.94 (0.79-1.11) | 0.459 |
| Stroke→Epilepsy | Unadjusted | Ref | Ref | 2.89 (1.35-6.17) | 0.006 | 2.06 (0.70-6.07) | 0.188 |
|  | Adjusted | Ref | Ref | 2.89 (1.35-6.17) | 0.006 | 2.06 (0.70-6.07) | 0.188 |
| Stroke→Delirium | Unadjusted | Ref | Ref | 1.22 (0.75-1.99) | 0.43 | 0.46 (0.19-1.10) | 0.081 |
|  | Adjusted | Ref | Ref | 1.28 (0.77-2.15) | 0.344 | 0.54 (0.21-1.40) | 0.206 |
| **1 years** | | | | | | | |
| Stroke→Depression | Unadjusted | Ref | Ref | 1.12 (0.89-1.40) | 0.327 | 0.70 (0.51-0.98) | 0.037 |
|  | Adjusted | Ref | Ref | 1.11 (0.88-1.41) | 0.373 | 0.71 (0.49-1.02) | 0.063 |
| Stroke→Disability | Unadjusted | Ref | Ref | 1.02 (0.93-1.11) | 0.691 | 0.83 (0.73-0.93) | 0.002 |
|  | Adjusted | Ref | Ref | 1.02 (0.93-1.12) | 0.61 | 0.85 (0.74-0.97) | 0.014 |
| Stroke→Epilepsy | Unadjusted | Ref | Ref | 1.02 (0.93-1.11) | 0.691 | 0.83 (0.73-0.93) | 0.002 |
|  | Adjusted | Ref | Ref | 1.14 (0.84-1.54) | 0.399 | 0.83 (0.53-1.29) | 0.399 |
| Stroke→Delirium | Unadjusted | Ref | Ref | 1.04 (0.83-1.32) | 0.723 | 0.50 (0.34-0.74) | <0.001 |
|  | Adjusted | Ref | Ref | 1.05 (0.82-1.35) | 0.673 | 0.60 (0.39-0.91) | 0.017 |

Note: Models adjusted for age, sex, ethnicity, education, Townsend deprivation index (TDI), smoking status, alcohol drink status, Body mass index (BMI), systolic blood pressure (SBP), diastolic blood pressure (DBP), sleep duration, metabolic syndrome (MetS) and healthy diet. Abbreviations: eGDR, Estimated glucose disposal rate; HR, Hazard ratio; CI, Confidence interval.

# Supplementary Table S13. Interaction terms for potential risk factors of eGDR associated with incident stroke and poststroke adverse outcomes

| **Characteristics** | **P for interaction** | | | | | | |
| --- | --- | --- | --- | --- | --- | --- | --- |
|  | **Stroke** | **Hemorrhagic stroke** | **Ischemic stroke** | **Poststroke depression** | **Poststroke disability** | **Poststroke epilepsy** | **Poststroke delirium** |
| Age | 0.024 | 0.751 | 0.195 | 0.134 | 0.652 | 0.688 | 0.528 |
| Sex | 0.035 | 0.757 | 0.152 | 0.163 | 0.639 | 0.303 | 0.096 |
| Ethnicity | 0.702 | 0.662 | 0.434 | 0.532 | 0.818 | 0.560 | 0.175 |
| Education | 0.224 | 0.200 | 0.452 | 0.613 | 0.908 | 0.874 | 0.391 |
| Smoking status | 0.783 | 0.064 | 0.815 | 0.089 | 0.738 | 0.789 | 0.010 |
| Alcohol intake status | 0.5566 | 0.474 | 0.547 | 0.034 | 0.090 | 0.006 | 0.016 |
| BMI | 0.027 | 0.502 | 0.016 | 0.041 | 0.182 | 0.003 | 0.108 |

Note: Models adjusted for age, sex, ethnicity, education, Townsend deprivation index (TDI), smoking status, alcohol drink status, Body mass index (BMI), systolic blood pressure (SBP), diastolic blood pressure (DBP), sleep duration, metabolic syndrome (MetS) and healthy diet.

# Supplementary Table S14. Multivariable linear regression for the associations between eGDR and inflammation biomarkers.

| **Inflammatory markers** | **Mean (SD)** | **eGDR** | | |
| --- | --- | --- | --- | --- |
|  |  | **Beta** | ***P* value** | ***P*_FDR__value** |
| C reactive protein, mg/L | 2.59 (4.35) | 0.006 | 0.001 | <0.001 |
| Lymphocyte percentage, % | 28.92 (7.49) | 0.017 | 0.001 | <0.001 |
| Lymphocyte count, 10^9^ cells/L | 1.97 (1.19) | 0.014 | 0.001 | <0.001 |
| Monocyte percentage, % | 7.06 (2.69) | -0.013 | 0.001 | <0.001 |
| Monocyte count, 10^9^ cells/L | 0.48 (0.28) | -0.013 | 0.001 | <0.001 |
| Platelet count, 10^9^ cells/L | 253.06 (59.99) | 0.010 | 0.001 | <0.001 |
| Neutrophil count, 10^9^ cells/L | 4.23 (1.42) | -0.005 | 0.001 | <0.001 |
| Neutrophil percentage, % | 60.87 (8.52) | -0.009 | 0.001 | <0.001 |
| SII | 599.49 (378.60) | -0.008 | 0.001 | <0.001 |
| NLR | 2.36 (1.28) | -0.015 | 0.001 | <0.001 |
| LMR | 4.64 (5.69) | 0.022 | 0.001 | <0.001 |
| WBC, 10^9^ cells/L | 6.88 (2.14) | -0.001 | 0.436 | 0.479 |
| PLR | 9.48 (4.54) | -0.006 | 0.001 | <0.001 |

Note: Models adjusted for age, sex, ethnicity, education, Townsend deprivation index (TDI), smoking status, alcohol drink status, Body mass index (BMI), systolic blood pressure (SBP), diastolic blood pressure (DBP), sleep duration, metabolic syndrome (MetS) and healthy diet; Abbreviations: LMR, lymphocyte-to-monocyte ratio; NLR, neutrophil-to-lymphocyte ratio; PLR, platelet-to-lymphocyte ratio; SII, systemic immune-inflammation index. WBC, White blood cell count; FDR, False discovery rate; SD, Standard Deviation.

# Supplementary Table S15. Longitudinal associations between the risk of incident stroke with inflammatory markers.

| **Inflammatory markers** | **Mean (SD)** | **Incident stroke** | | | |
| --- | --- | --- | --- | --- | --- |
|  |  | **HR** | **95%CI** | ***P* value** | ***P*_FDR__value** |
| C reactive protein, mg/L | 2.59 (4.35) | 1.10 | 1.07-1.12 | <0.001 | <0.001 |
| Lymphocyte percentage, % | 28.92 (7.49) | 0.94 | 0.91-0.94 | <0.001 | <0.001 |
| Lymphocyte, 10^9^ cells/L | 1.97 (1.19) | 0.97 | 0.95-0.99 | 0.002 | 0.003 |
| Monocyte percentage, % | 7.06 (2.69) | 1.02 | 1.01-1.04 | 0.013 | 0.014 |
| Monocyte, 10^9^ cells/L | 0.48 (0.28) | 1.07 | 1.05-1.09 | <0.001 | <0.001 |
| Platelet, 10^9^ cells/L | 253.06 (59.99) | 1.02 | 0.99-1.04 | 0.062 | 0.072 |
| Neutrophil, 10^9^ cells/L | 4.23 (1.42) | 1.07 | 1.05-1.09 | <0.001 | <0.001 |
| Neutrophil percentage, % | 60.87 (8.52) | 1.04 | 1.02-1.06 | <0.001 | <0.001 |
| SII | 599.49 (378.60) | 1.07 | 1.05-1.09 | <0.001 | <0.001 |
| NLR | 2.36 (1.28) | 1.07 | 1.05-1.09 | <0.001 | <0.001 |
| LMR | 4.64 (5.69) | 0.92 | 0.91-0.94 | <0.001 | <0.001 |
| WBC, 10^9^ cells/L | 6.88 (2.14) | 1.07 | 1.05-1.09 | <0.001 | <0.001 |
| PLR | 9.48 (4.54) | 1.08 | 1.06-1.09 | <0.001 | <0.001 |

Note: Models adjusted for age, sex, ethnicity, education, Townsend deprivation index (TDI), smoking status, alcohol drink status, Body mass index (BMI), systolic blood pressure (SBP), diastolic blood pressure (DBP), sleep duration, metabolic syndrome (MetS) and healthy diet; Abbreviations: eGDR, Estimated glucose disposal rate; HR, Hazard ratio; CI, Confidence interval; LMR, lymphocyte-to-monocyte ratio; NLR, neutrophil-to-lymphocyte ratio; PLR, platelet-to-lymphocyte ratio; SII, systemic immune-inflammation index. WBC, White blood cell count; FDR, False discovery rate; SD, Standard Deviation.

# Supplementary Table S16. The mediation effects of Inflammatory markers in the association between eGDR with stroke at baseline.

| **Inflammatory markers** | **Decrements in eGDR** | | | | | | | | | | | | |
| --- | --- | --- | --- | --- | --- | --- | --- | --- | --- | --- | --- | --- | --- |
|  | **Direct effect** | | | **Indirect effect** | | | **Total effect** | | | **Mediation proportion** | | | |
|  | **β** | ***P* value** | ***P*_FDR_** | **β** | ***P* value** | ***P*_FDR_** | **β** | ***P* value** | ***P*_FDR_** | **β** | **95%CI** | ***P* value** | ***P*_FDR_** |
| WBC, 10^9^ cells/L | 1.32E+01 | <2E-16 | 2.60E-15 | 1.71E-02 | 0.02 | 0.028 | 1.33E+01 | <2E-16 | 2.60E-15 | 1.29E-03 | 0.02%-0.30% | 0.024 | 0.031 |
| Platelet count, 10^9^ cells/L | 1.33E+01 | <2E-16 | 2.60E-15 | -2.65E-04 | 0.90 | 1.00 | 1.33E+01 | <2E-16 | 2.60E-15 | -1.99E-05 | -0.03%-0.00% | 0.900 | 1.00 |
| C reactive protein, mg/L | 1.33E+01 | <2E-16 | 2.60E-15 | -1.59E-02 | 0.11 | 0.119 | 1.33E+01 | <2E-16 | 2.60E-15 | -1.20E-0 | -0.26%-0.00% | 0.11 | 0.119 |
| Neutrophil, 10^9^ cells/L | 1.32E+01 | <2E-16 | 2.60E-15 | 4.71E-02 | <2E-16 | 2.60E-15 | 1.33E+01 | <2E-16 | 2.60E-15 | 3.55E-03 | 0.23%-0.50% | <2E-16 | 2.60E-15 |
| Neutrophil % | 1.32E+01 | <2E-16 | 2.60E-15 | 3.37E-02 | <2E-16 | 2.60E-15 | 1.33E+01 | <2E-16 | 2.60E-15 | 2.54E-03 | 0.08%-0.40% | <2E-16 | 2.60E-15 |
| Lymphocyte, 10^9^ cells/L | 1.33E+01 | <2E-16 | 2.60E-15 | 2.43E-02 | <2E-16 | 2.60E-15 | 1.33E+01 | <2E-16 | 2.60E-15 | 1.83E-03 | 0.01%-0.30% | 0.020 | 0.028 |
| Lymphocyte % | 1.32E+01 | <2E-16 | 2.60E-15 | 1.19E-02 | 0.024 | 0.031 | 1.33E+01 | <2E-16 | 2.60E-15 | 8.94E-03 | 0.66%-1.00% | <2E-16 | 2.60E-15 |
| Monocyte, 10^9^ cells/L | 1.32E+01 | <2E-16 | 2.60E-15 | 4.05E-02 | <2E-16 | 2.60E-15 | 1.33E+01 | <2E-16 | 2.60E-15 | 3.05E-03 | 0.18%-0.40% | <2E-16 | 2.60E-15 |
| Monocyte % | 1.33E+01 | <2E-16 | 2.60E-15 | 1.10E-02 | 0.024 | 0.031 | 1.33E+01 | <2E-16 | 2.60E-15 | 8.27E-04 | 0.01%-0.20% | 0.024 | 0.031 |
| NLR | 1.32E+01 | <2E-16 | 2.60E-15 | 9.34E-02 | <2E-16 | 2.60E-15 | 1.33E+01 | <2E-16 | 2.60E-15 | 7.03E-03 | 0.50%-1.00% | <2E-16 | 2.60E-15 |
| LMR | 1.32E+01 | <2E-16 | 2.60E-15 | 1.07E-01 | <2E-16 | 2.60E-15 | 1.33E+01 | <2E-16 | 2.60E-15 | 8.08E-03 | 0.58%-1.00% | <2E-16 | 2.60E-15 |
| PLR | 1.32E+01 | <2E-16 | 2.60E-15 | 7.70E-02 | <2E-16 | 2.60E-15 | 1.33E+01 | <2E-16 | 2.60E-15 | 5.78E-03 | 0.39%-1.00% | <2E-16 | 2.60E-15 |
| SII | 1.32E+01 | <2E-16 | 2.60E-15 | 7.56E-02 | <2E-16 | 2.60E-15 | 1.33E+01 | <2E-16 | 2.60E-15 | 5.68E-03 | 0.37%-1.00% | <2E-16 | 2.60E-15 |

Note: Models adjusted for age, sex, ethnicity, education, Townsend deprivation index (TDI), smoking status, alcohol drink status, Body mass index (BMI), systolic blood pressure (SBP), diastolic blood pressure (DBP), sleep duration, metabolic syndrome (MetS) and healthy diet. Note: Abbreviations: LMR, lymphocyte-to-monocyte ratio; NLR, neutrophil-to-lymphocyte ratio; PLR, platelet-to-lymphocyte ratio; SII, systemic immune-inflammation index. WBC, White blood cell count; FDR, False discovery rate; β, Standardized Regression Coefficient.

# Supplementary Figure S1. The directed acyclic graph (DAG) illustrates the relationship between the exposures (eGDR), the mediators, the outcome (Stroke), and the covariates included in the analyses. The direction of arrows in the acyclic graph was based on previous literature, The DAG was drawn using <http://www.dagitty.net/>.


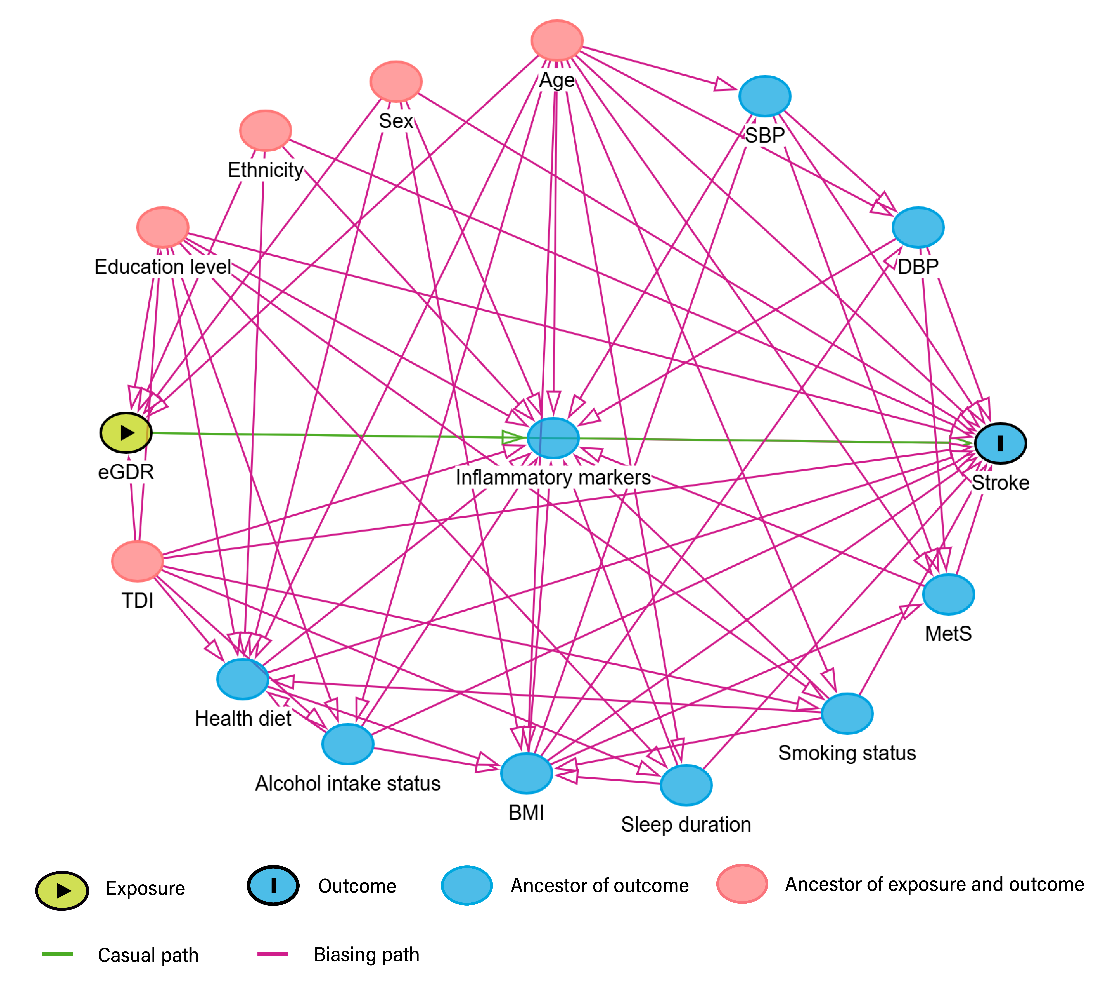


# Supplementary Figure S2. Associations between eGDR and incidence of stroke, with separate analyses for sex and age.


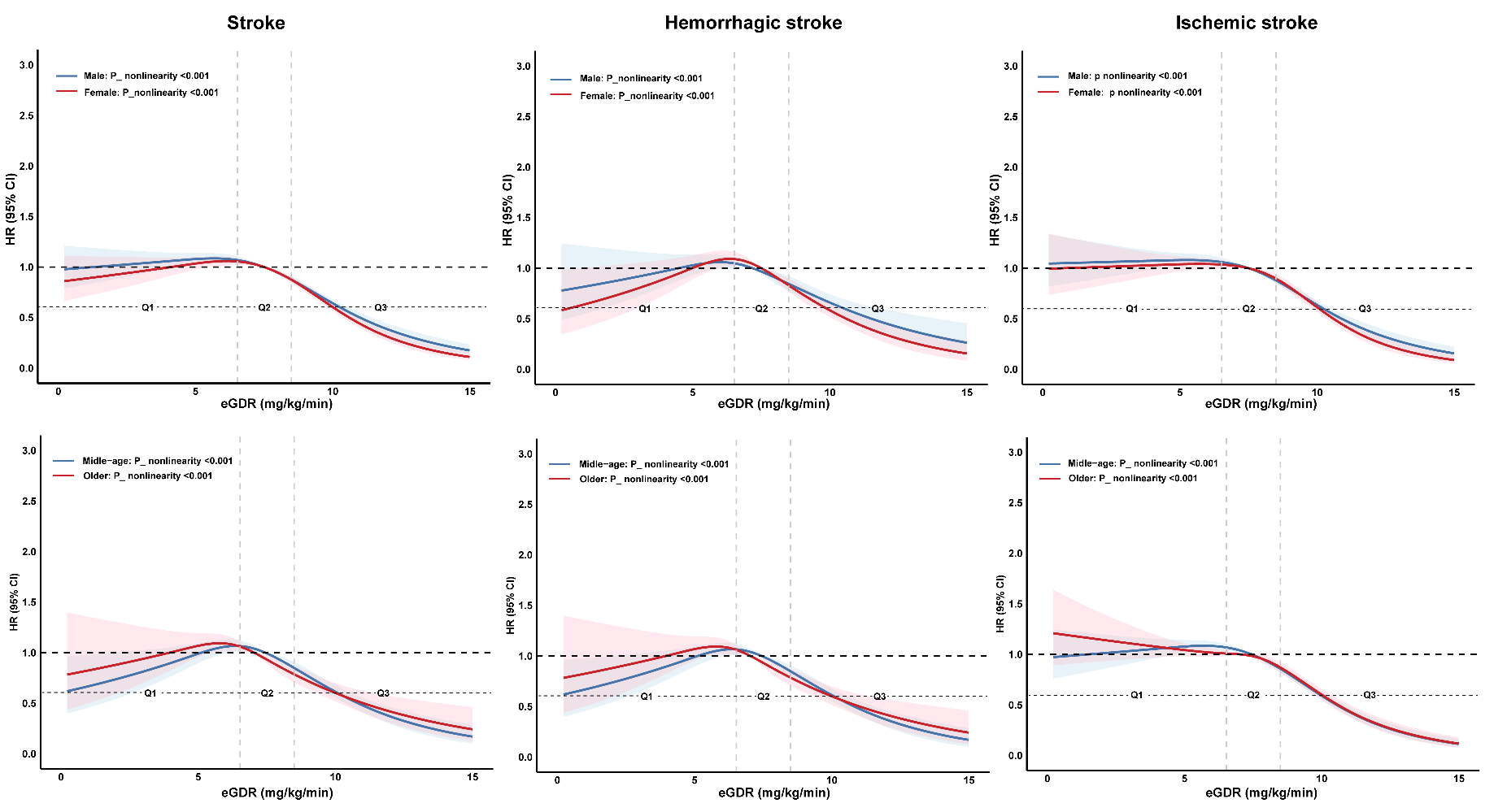


Note: Models adjusted for age, sex, ethnicity, education, Townsend deprivation index (TDI), smoking status, alcohol drink status, Body mass index (BMI), systolic blood pressure (SBP), diastolic blood pressure (DBP), sleep duration, metabolic syndrome (MetS) and healthy diet; male/mild-age (blue) and female/older (red).

# Supplementary Figure S3. Subgroup analysis of eGDR with incidence of stroke.


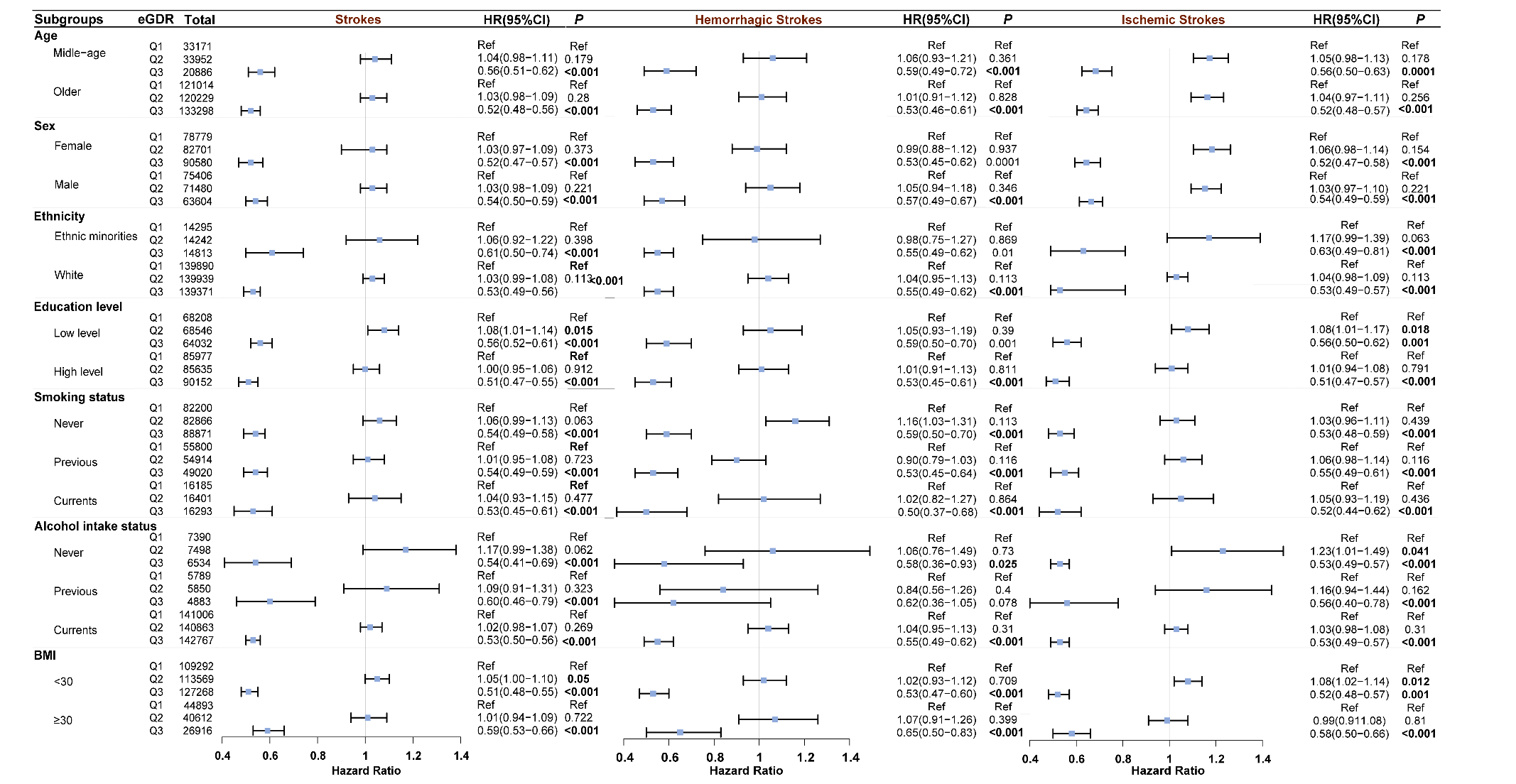


Note: Models adjusted for age, sex, ethnicity, education, Townsend deprivation index (TDI), smoking status, alcohol drink status, Body mass index (BMI), systolic blood pressure (SBP), diastolic blood pressure (DBP), sleep duration, metabolic syndrome (MetS) and healthy diet.

# Supplementary Figure S4. Subgroup analysis of eGDR with incidence of poststroke adverse outcomes.


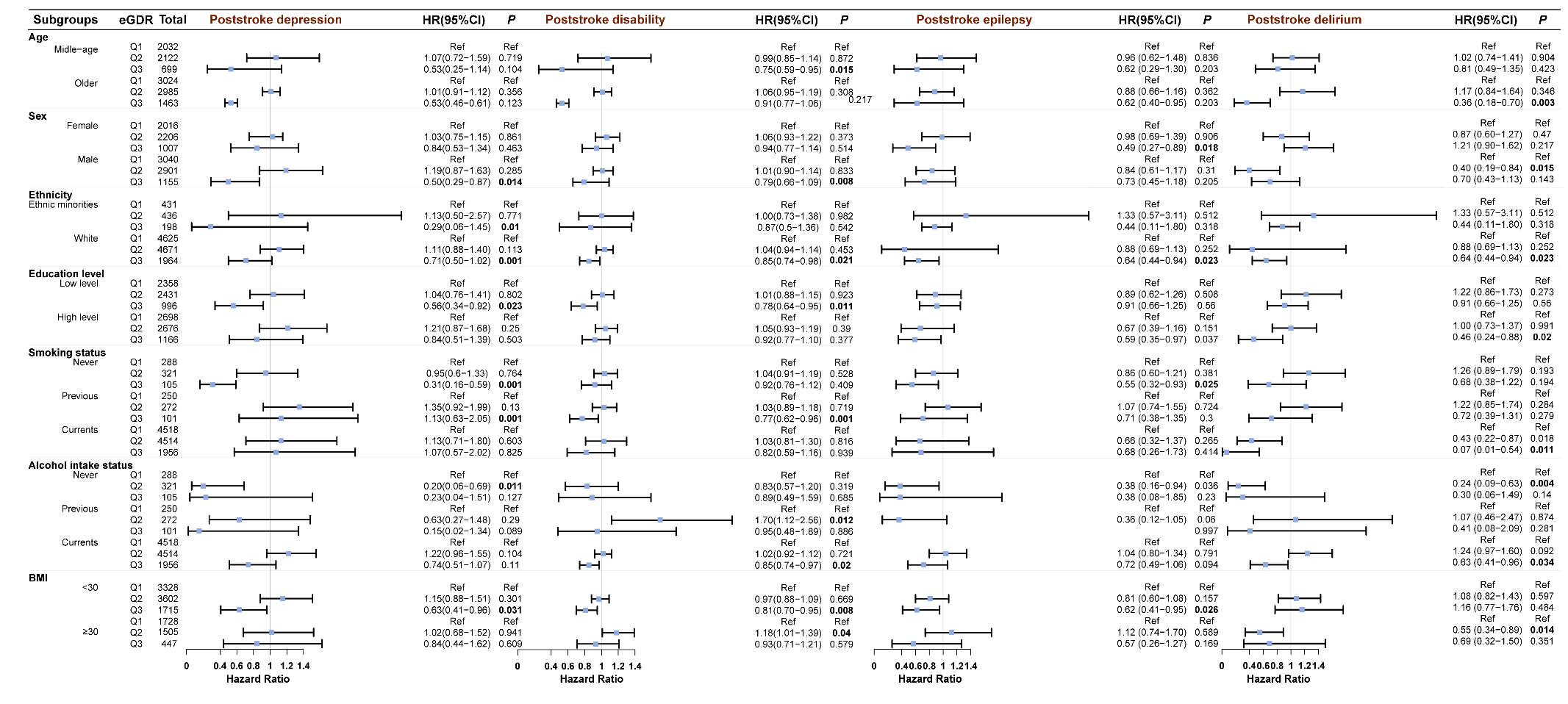


Note: Models adjusted for age, sex, ethnicity, education, Townsend deprivation index (TDI), smoking status, alcohol drink status, Body mass index (BMI), systolic blood pressure (SBP), diastolic blood pressure (DBP), sleep duration, metabolic syndrome (MetS) and healthy diet.

# Supplementary Figure S5. Heatmap of associations between blood inflammatory markers with eGDR and incident stroke risks.


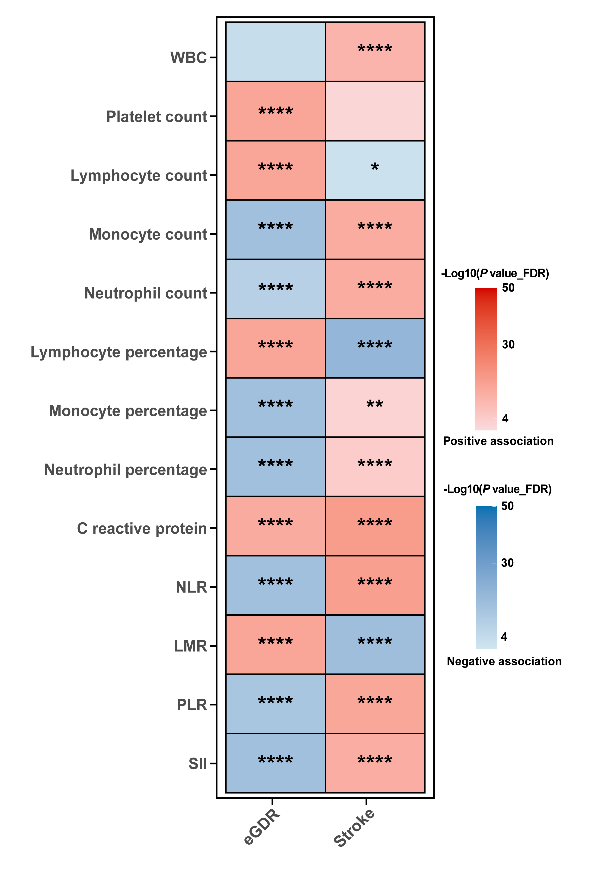


Note: FDR-adjusted P values < 0.05 indicate statistically associations, as determined based on multiple-adjusted linear models (eGDR as independent variables, inflammatory markers as dependent variables) or Cox proportional hazard models (inflammatory markers as independent variables, incident stroke as dependent variables). ^****^P values < 0.0001, ^***^P values < 0.001, ^**^P values < 0.01, ^*^P values < 0.05. Models adjusted for age, sex, ethnicity, education, Townsend deprivation index (TDI), smoking status, alcohol drink status, Body mass index (BMI), systolic blood pressure (SBP), diastolic blood pressure (DBP), sleep duration, metabolic syndrome (MetS) and healthy diet.

# Supplementary Figure S6. Differences in inflammatory markers between the lowest (Q1) and highest (Q3) eGDR tertiles.


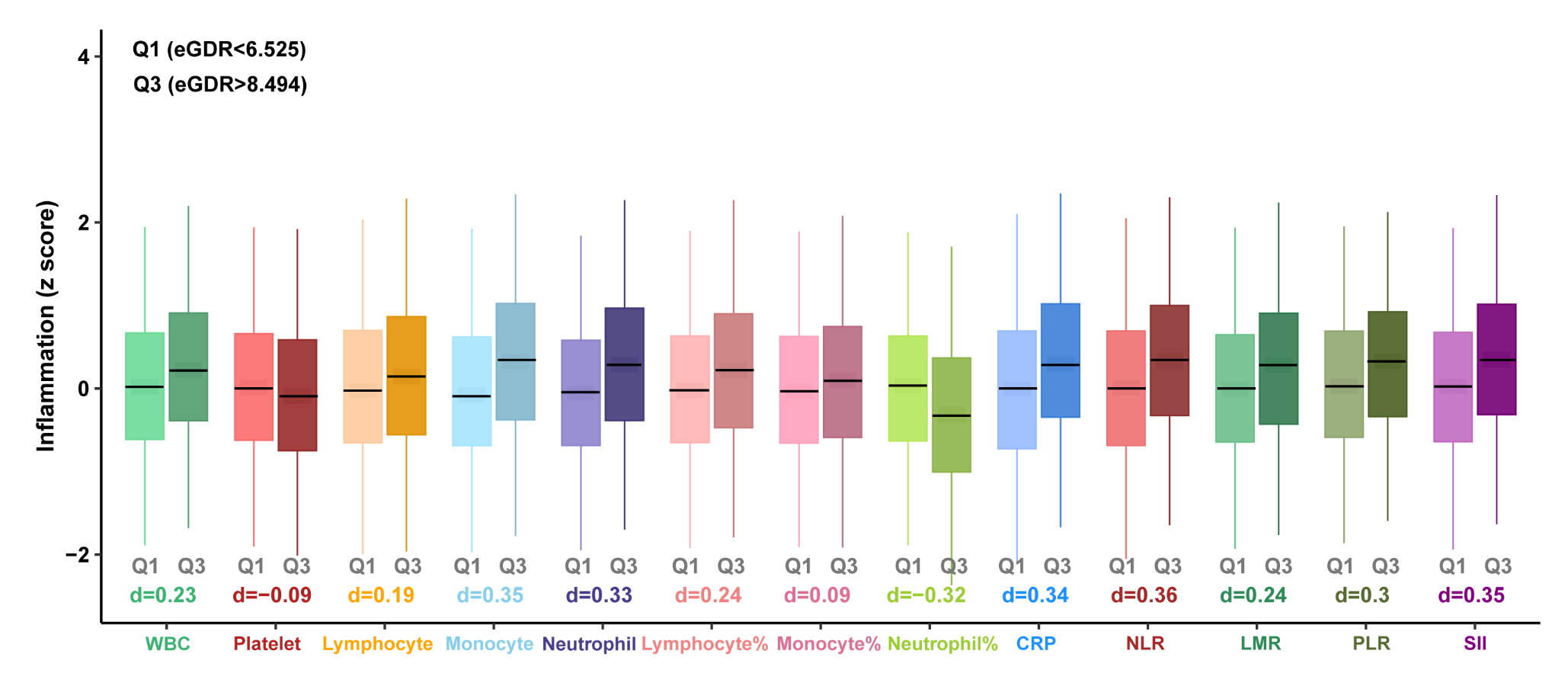


Note: In a box-and-line plot with the center line as the median and the box constrained to the upper and lower quartiles. Size of the bar and the internal center represent mean Cohen’s *d*. The error bar represents the corresponding 95% CI. Models adjusted for age, sex, ethnicity, education, Townsend deprivation index (TDI), smoking status, alcohol drink status, Body mass index (BMI), systolic blood pressure (SBP), diastolic blood pressure (DBP), sleep duration, metabolic syndrome (MetS) and healthy diet.
